# Supplementary material for: Full-color processible afterglow organic small molecular glass
Source: Nat Commun. 2025 May 15;16:4526. doi: 10.1038/s41467-025-59787-y (PMC12081614; doi:10.1038/s41467-025-59787-y)
Supplement: Supplementary file 1 — Supplementary Information [file 41467_2025_59787_MOESM1_ESM.pdf]

`

Supplementary Information

Full-color processible afterglow organic small molecular glass

*Yufeng Xue[1], Zongliang Xie[1,2], Zheng Yin[1] ,Yincai Xu[1] and Bin Liu[1,2]**

[1]Department of Chemical and Biomolecular Engineering, National University of Singapore, Singapore. 4 Engineering Drive 4, Singapore, 117585.

[2]Institute for Functional Intelligent Materials, National University of Singapore, Singapore. 4 Science Drive 2, Singapore, 117544.

* E-mail: cheliub@nus.edu.sg

`

Supplementary Methods

General methods

Proton, carbon and phosphorus nuclear magnetic resonance (^{1}H NMR, ^{13}C NMR, ^{31}P NMR) spectra for the TTPO were attained from a Bruker ARX 400 NMR spectrometer with Chloroform-*d* ($CDCl_3$). The Kissinger analysis for TTPO activation energy from glassy to crystalline state was determined by using a differential scanning calorimeter (PerkinElmer 8000 DSC). Thermogravimetric analysis of TTPO was performed by using a thermogravimetric analyzer (Shimadzu DTG-60 AH). The viscosity of TTPO supercool liquid was measured by using a rheometer (Anton Paar MCR-9). UV–vis absorption spectrum of each compound was obtained using a UV–vis spectrometer (Shimadzu UV-2600). Delayed emission spectra were collected with the Ocean Optic QE 65 Pro spectrometer. Photoluminescence (PL) spectra and phosphorescence lifetimes were recorded with an Edinburgh Instruments Spectrofluorometer (FLS 1000). The quantum yields of the doped systems were measured by FLS 1000 Spectrofluorometer with an integrating sphere. The crystal structure of TTPO is available from the CCDC database (CCDC number: 2425812).

Theoretical Calculations

Density functional theory (DFT) computations, encompassing geometrical optimization and electronic properties evaluations at ground states, were conducted using the Gaussian 09 software package at the B3LYP/6-311g(d) level[1]. The energy

levels of the excited states were computed using the time-dependent DFT (TDDFT) method at the same B3LYP/6-311g(d) level. The spin-orbit coupling (SOC) matrix elements between singlet and triplet excited states were calculated by ORCA 5.0.4 using the B3LYP/G method with DKH2 DKH-def2-TZVP basis sets[2-4]. Analysis was performed using Multiwfn 3.8[5].

Fiber fabrication and hot-pressing methods

Stamp: TTPO (2.0 g) and guest (20 mg) were melted at 170 °C in a spoon. When the supercooled liquid reached 80 °C, an NUS mold stamp was pressed onto it. After removing the mold, an NUS afterglow glass stamp was obtained.

Fiber: TTPO (1.0 g) and guest (10 mg) were melted at 170 °C in a dish. The molten liquid was quickly transferred onto an 80 °C hot plate, from which a meter-scale fiber could be drawn from the supercooled liquid.

PMMA sample preparation

Dopants@PMMA doping systems were prepared according to the reported literature[6]. For the 9-DT@PMMA system, a concentration of 2 wt% was used to replicate the experiment, while all other comparisons were conducted at 1 wt%. 100mg of PMMA (Mw: 350 kDa) and 1 mg of the guest compound were dissolved in 1 mL of dichloromethane and syringe filtered. The quartz substrate was set to 50 ℃, and the mixed solution was drop-cast onto a quartz substrate. After completely evaporation of the solvent, the samples were subjected to thermal annealing at 140 ℃ for 1 h, resulting in the preparation of the dopants@PMMA film.

Materials and syntheses

Synthetic routes for tri(2-methylphenyl) phosphine oxide. 1-Methyl 2-amino-3-methoxybenzoate (TCI, > 98%), coumarin 1 (Sigma, 99%), 7*H*-benzo[c]carbazole (TCI, > 98%), tris (1-naphthyl) phosphine (TCI, > 98%), 1,8-naphthalic anhydride (TCI, > 98%), 5*H*-benzo[*b*]carbazole (TCI, > 95%), pyrene (TCI, > 98%), pyrene carboxylic acid (TCI, > 97%) and 2,6-dibromo-4,4-difluoro-1,3,5,7-tetramethyl-8-phenyl-4-bora-3a,4a-diaza-s-indacene (BLDPharm, > 95%) were purchased from BLDPharm, TCI and sigma. PMMA (Mw: 350 kDa) was purchased from Sigma-Aldrich. All the products were purified by column chromatography before use.

Synthesis of tri(2-methylphenyl) phosphine oxide (TTPO)

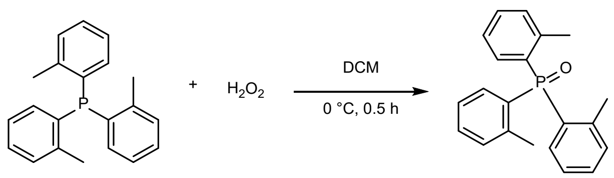

Tri(2-methylphenyl) phosphine (100.0 g, 328 mmol) was dissolved in 500 mL of dichloromethane (DCM), and the flask was placed in an ice bath at 0°C under vigorous stirring. Hydrogen peroxide (H_2O_2, 27 mL, 394 mmol, 35% in water) was added dropwise over 5 minutes, followed by continued stirring for an additional 30 minutes until complete consumption of tri(2-methylphenyl) phosphine was confirmed by TLC. The reaction mixture was carefully quenched by dropwise addition into a saturated sodium sulfite solution at 0°C to neutralize excess H_2O_2. The resulting mixture was extracted with dichloromethane three times. The combined organic layers were concentrated under reduced pressure to afford the crude product as a white powder. The product was purified by silica-gel column chromatography followed by recrystallization, yielding TTPO as a white solid with 99% yield (104.5 g). TTPO ^{1}H NMR (400 MHz, $CDCl_3$) δ 7.41–7.44 (m, 3H), 7.29–7.32 (3, m), 7.06–7.17 (m, 6H), 2.49 (s, 9H). ^{13}C NMR (100 MHz, $CDCl_3$) δ 143.52 (d, J = 8 Hz), 132.92 (d, J = 13 Hz), 132.05 (d, J = 10 Hz), 131.87 (d, J = 3 Hz), 130.5 (d, J = 101 Hz), 125.51 (d, J = 12 Hz). ^{31}P NMR (162 MHz, $CDCl_3$) 37.3.

Supplementary Figures

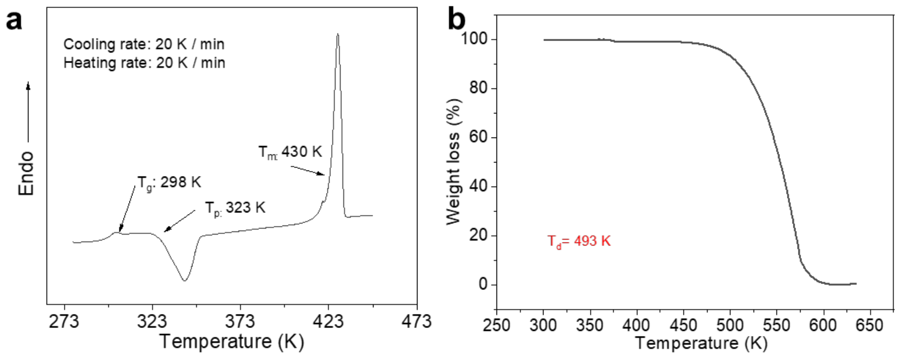

Supplementary Fig. 1 | a) Differential scanning calorimetry (DSC) measurement to determine the glass transition temperature, crystallization temperature (onset) and melting point. b) Thermogravimetry Analysis (TGA) curve of TTPO.

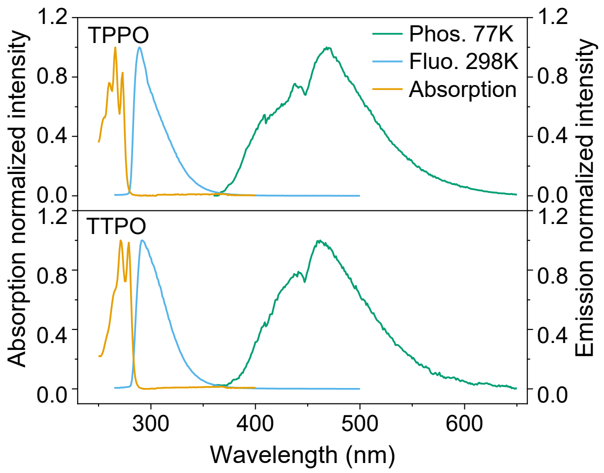

Supplementary Fig. 2 | Absorption and fluorescence spectra of TPPO and TTPO, and phosphorescence spectra of TPPO and TTPO powders at 77 K. Absorption and fluorescence spectra were collected under 254 nm excitation. Phosphorescence spectra were collected under 254 nm excitation.

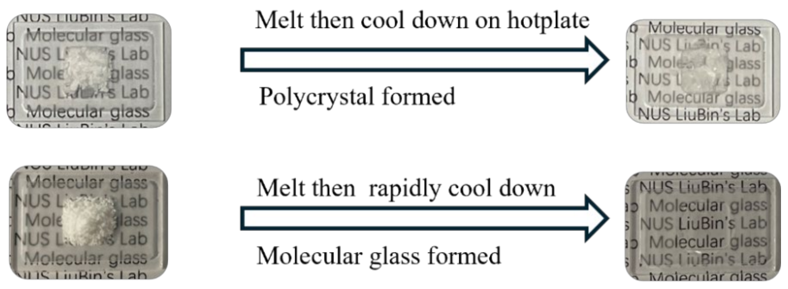

Supplementary Fig. 3 | The effect of vitrification on glass formation.

`

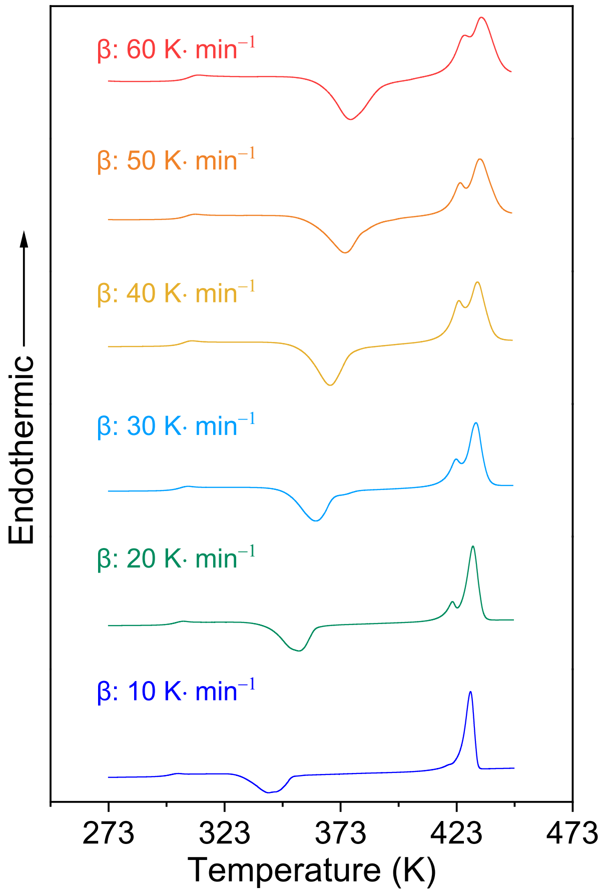

Supplementary Fig. 4 | DSC curves of TTPO under N_2 atmosphere at 20 K· min^{-1} cooling rate and different heating rate.

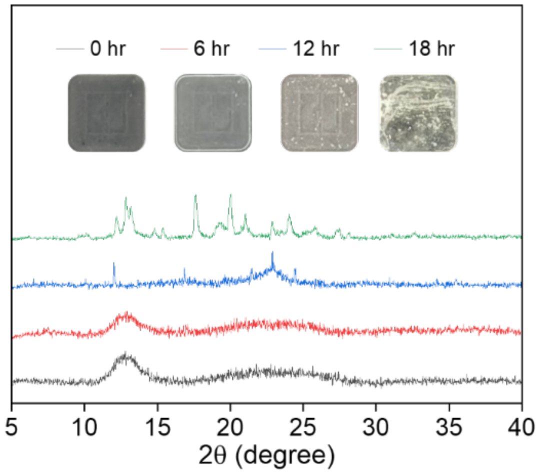

Supplementary Fig. 5 | Time-dependent p-XRD measurements of TTPO films.

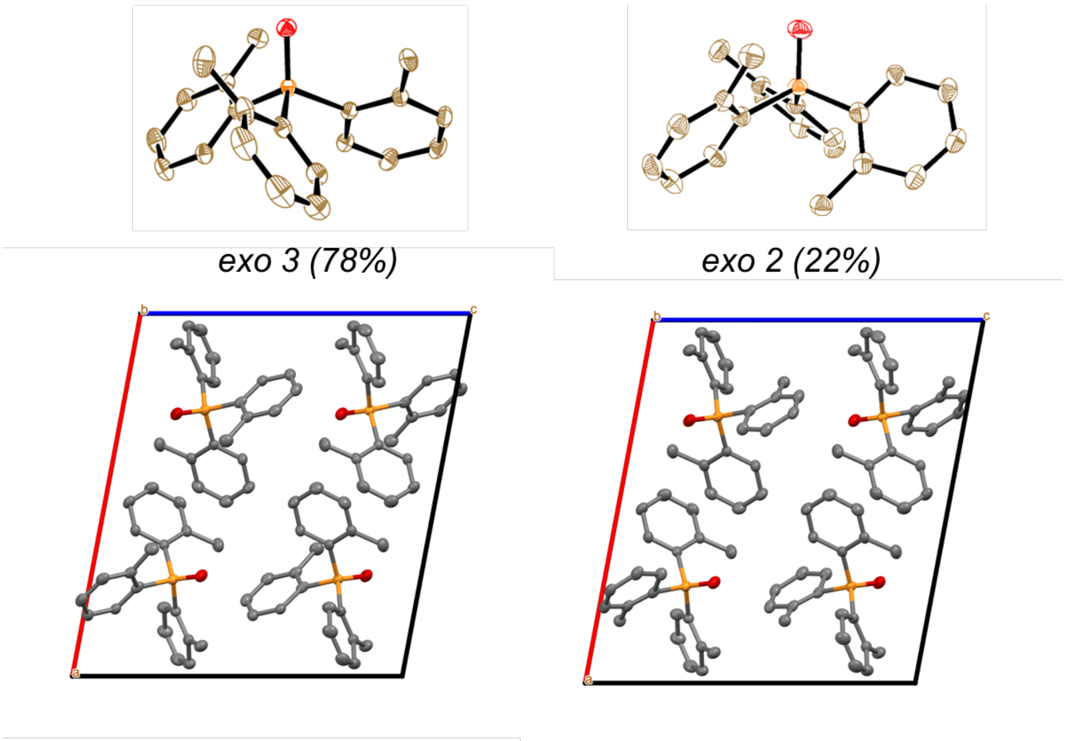

Supplementary Fig. 6 | Crystal information of TTPO. TTPO was crystallized from triethylamine, forming a monoclinic crystal system with space group $P2_1/c$. The asymmetric unit comprises a TTPO with molecular formula of $C_{21}H_{21}OP$. One of the *o*-tolyl groups is disordered into two positions with an occupancy ratio of 78:22 which corresponds to the *exo3* and *exo2* conformations. The crystal structure has been deposited on the CCDC website under the deposition number 2425812.

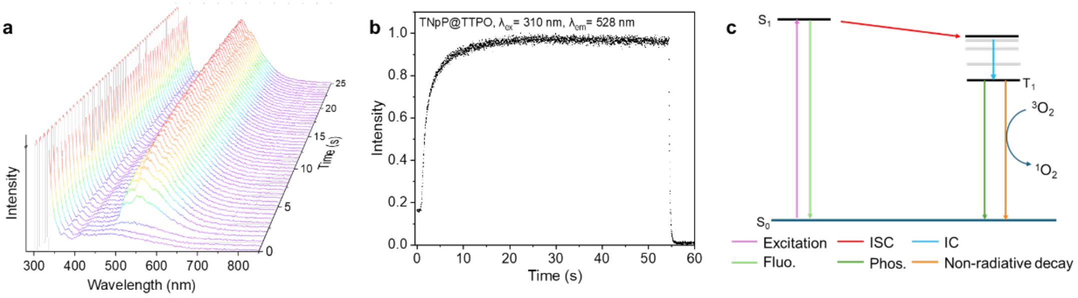

Supplementary Fig. 7 | a) Time-dependent PL spectra of the TNpP@TTPO doping system (excited at 310 nm to minimize interference from the excitation source). b) Time-dependent emission intensity at 528 nm. c) Schematic illustration of the photoactivation process.

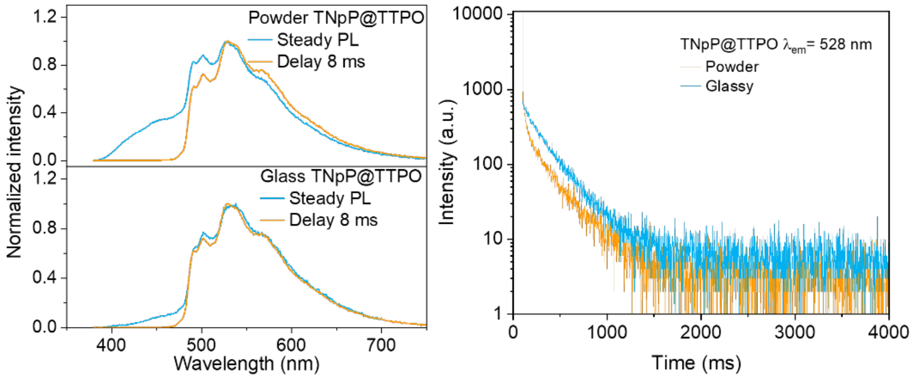

Supplementary Fig. 8 | PL, delay spectra, and lifetime profile of TNpP@TTPO in glassy state and powder state (365 nm excitation).

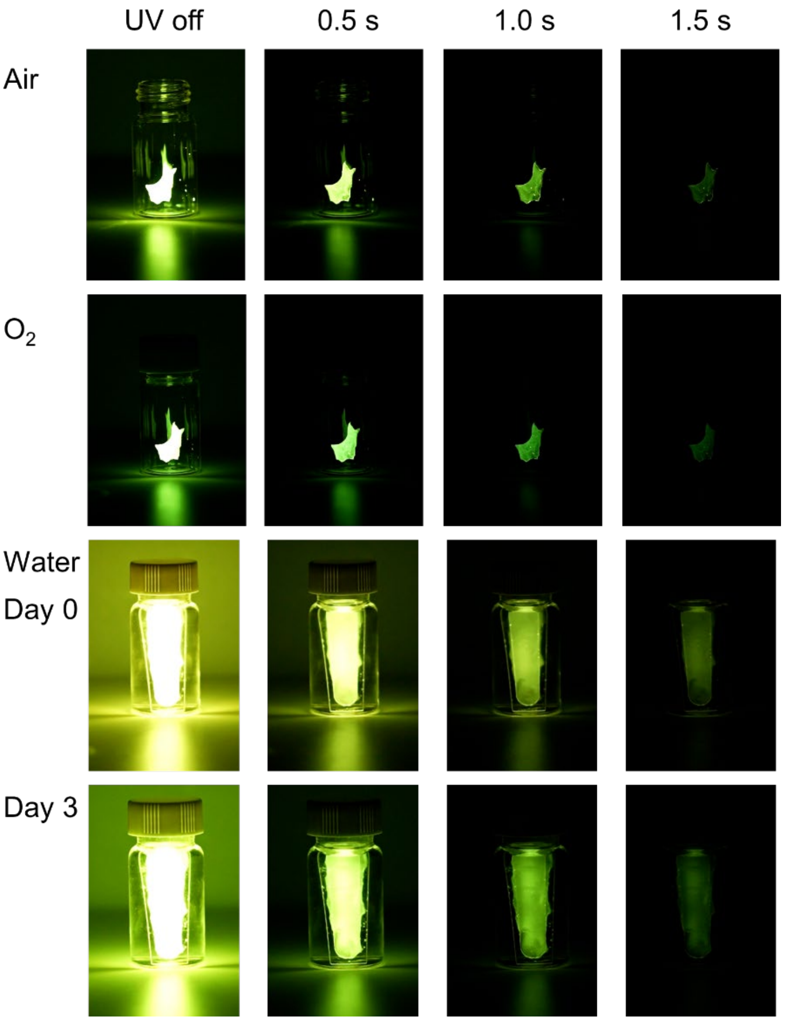

Supplementary Fig. 9 | Afterglow properties of TNpP@TTPO in water and oxygen.

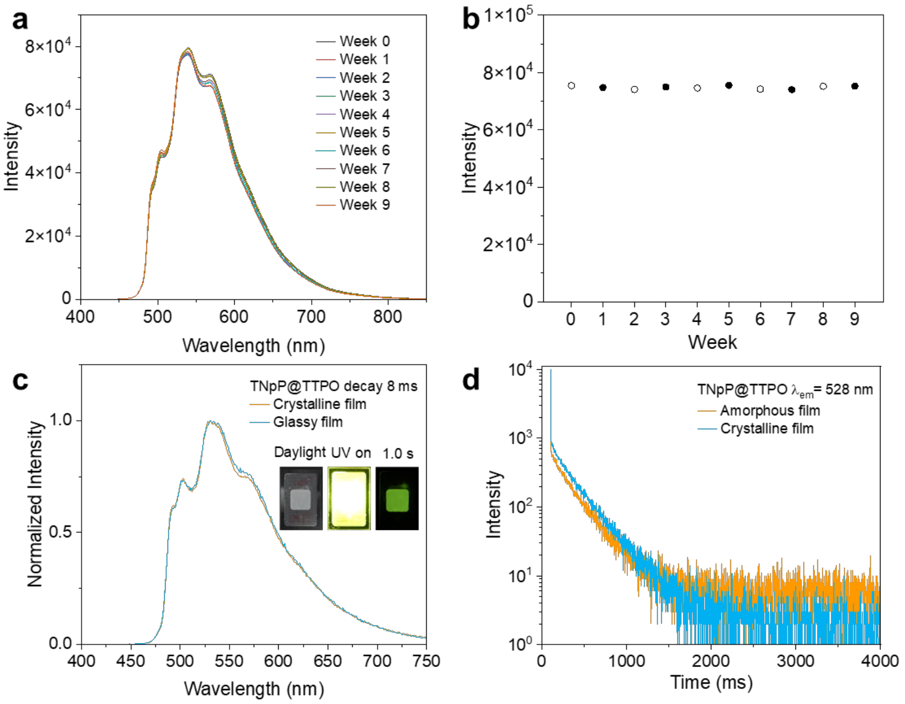

Supplementary Fig. 10 | a) Phosphorescence spectra at different weeks. b) Phosphorescence intensity over 9 weeks. c) The decay 8 ms spectra and photo of TNpP@TTPO glassy and crystalline films. d) Phosphorescence lifetime of TNpP@TTPO glassy and crystalline films at 528 nm (Camara setting: ISO 400 for daylight imaging, ISO 5000 for phosphorescence imaging).

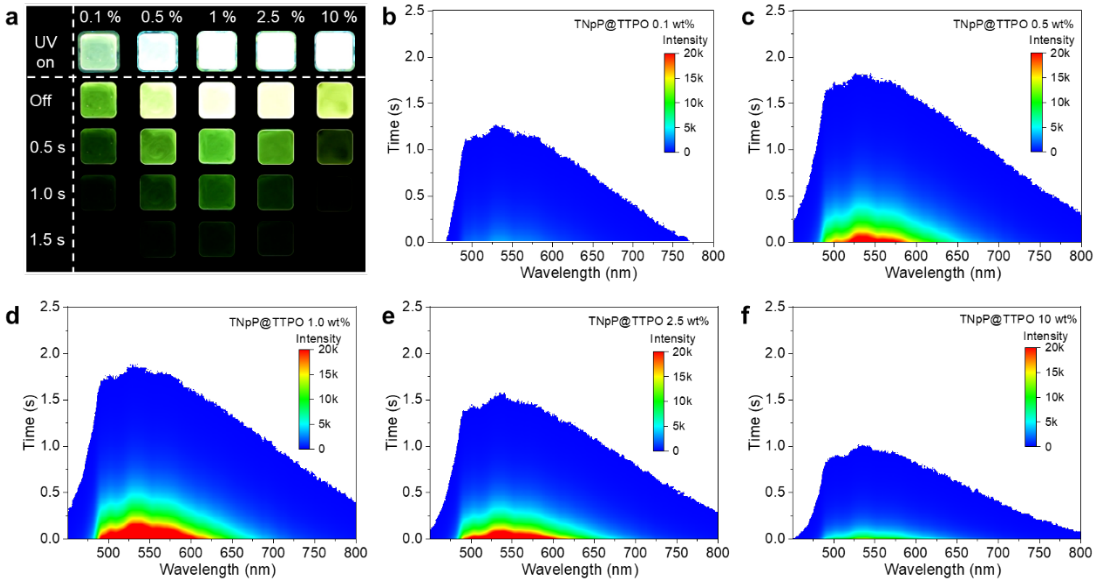

Supplementary Fig. 11 | Afterglow photographs and time-resolved phosphorescence spectra of TNpP@TTPO with varying doping ratios.

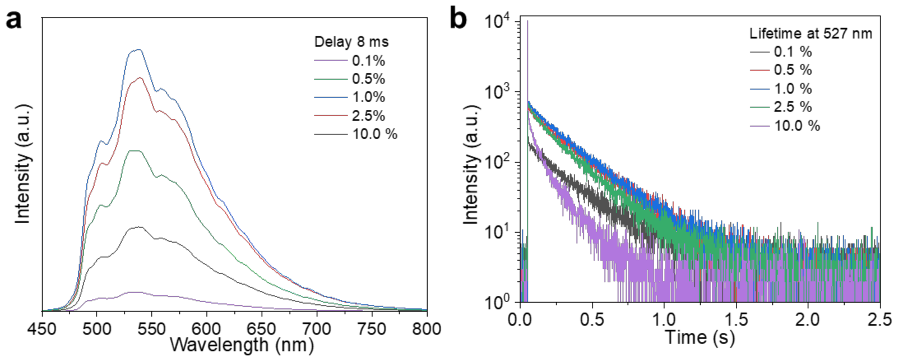

Supplementary Fig. 12 | Dealy 8 ms and decay profile at 527 nm of TNpP@TTPO with varying doping ratios.

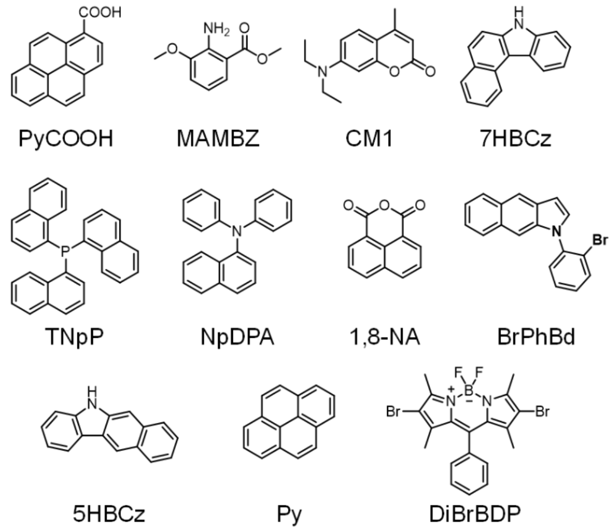

Supplementary Fig. 13 | Chemical structures of guests.

`

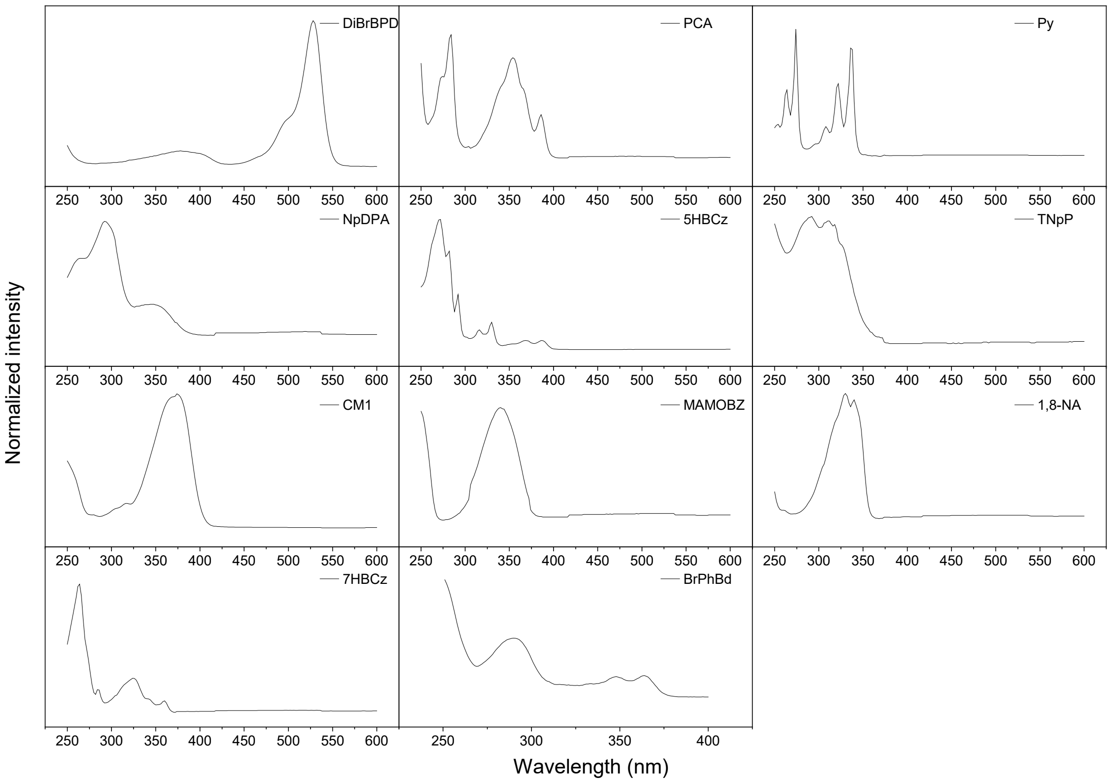

Supplementary Fig. 14 | Absorption spectra of guests in DCM ([c] = 10^{-5} M).

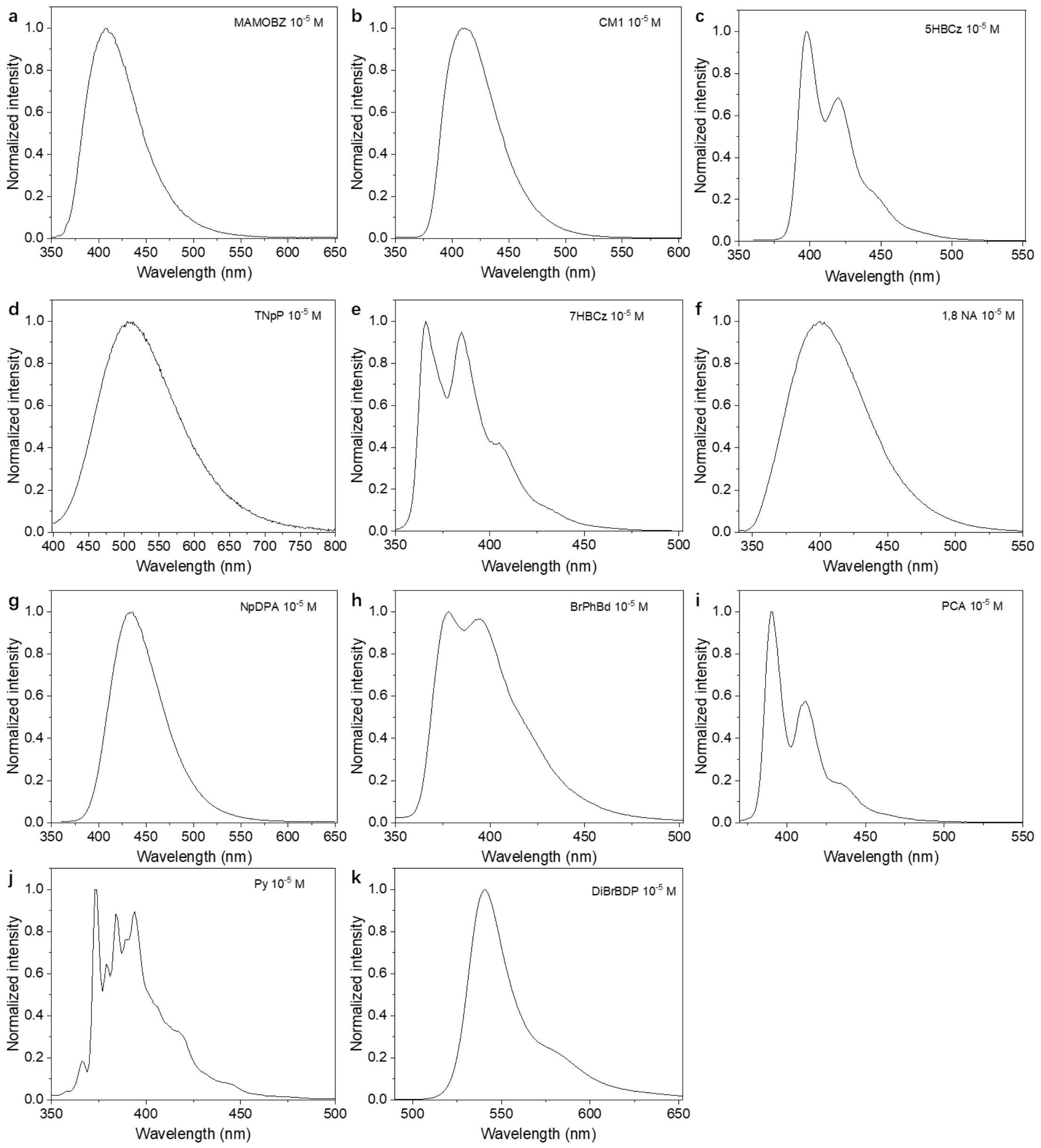

Supplementary Fig. 15 | Fluorescence spectra of guests in toluene at room temperature (310 nm excitation, [*c*] = 10^{-5} M)

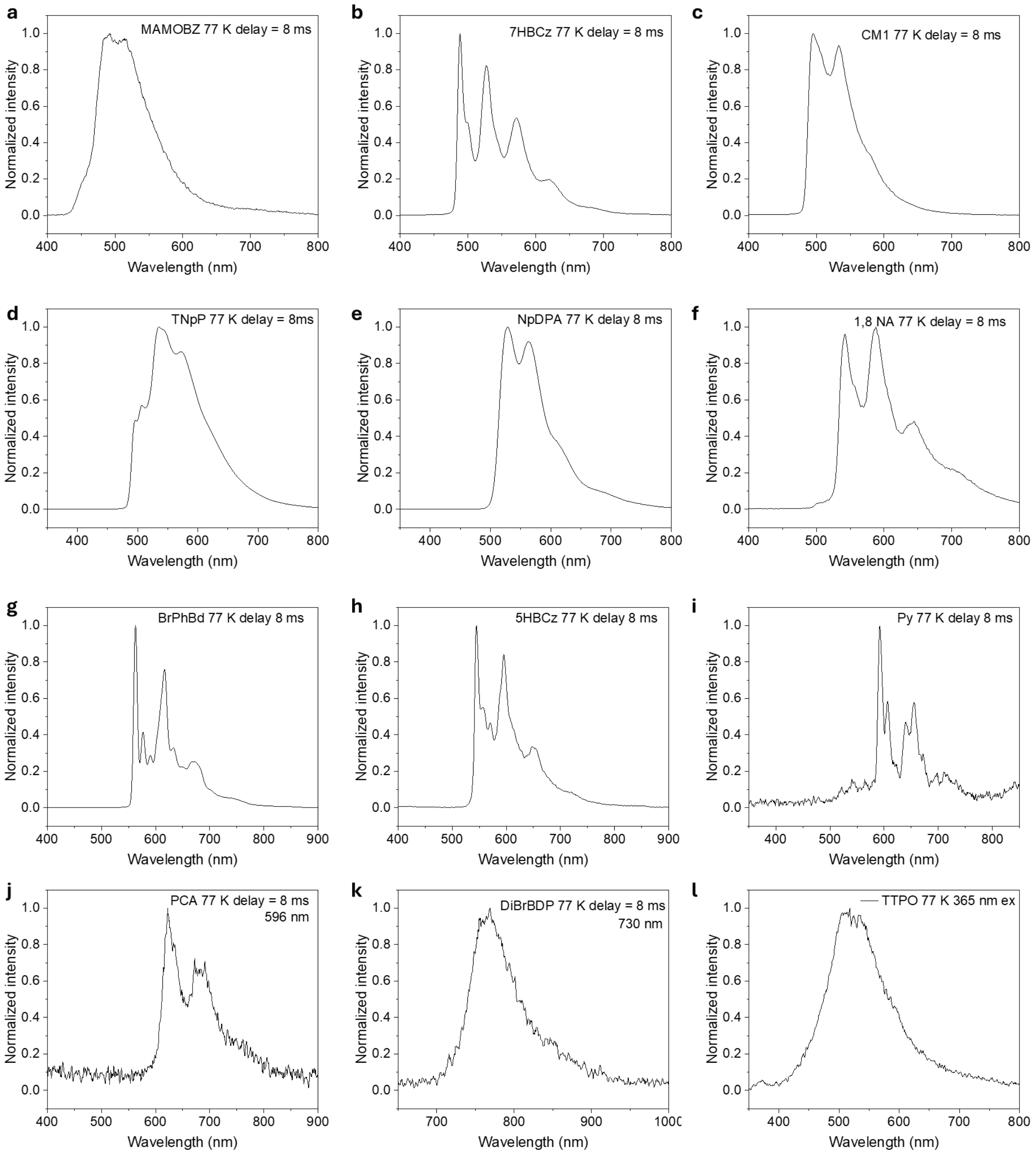

Supplementary Fig. 16 | Delay 8 ms spectra of guests in toluene at 77 K (365 nm excitation, [c] = 10^{-3} M).

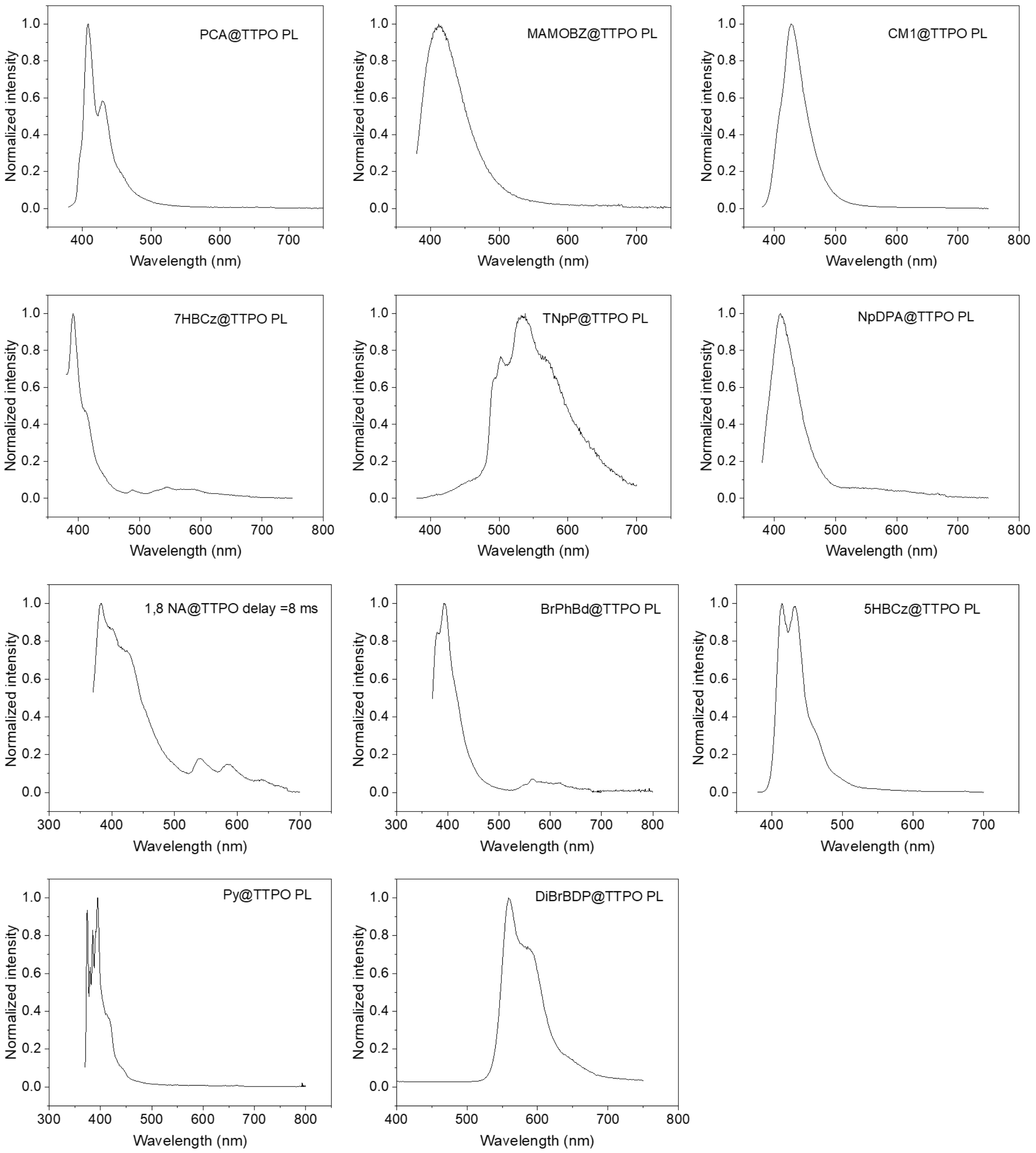

Supplementary Fig. 17 | PL spectra of guest@TTPO doping system (365 nm excitation).

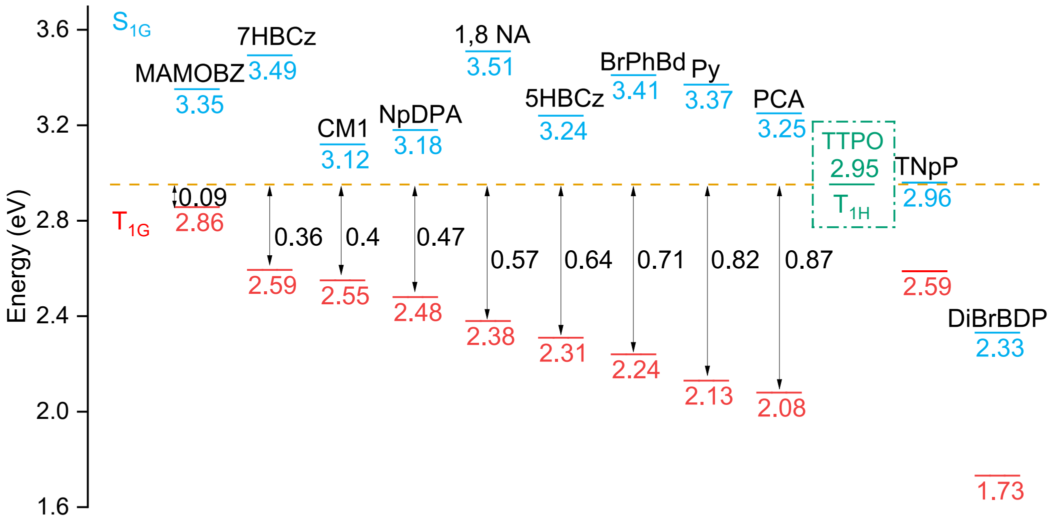

Supplementary Fig. 18 | Singlet and triplet energy distribution of guests and host.

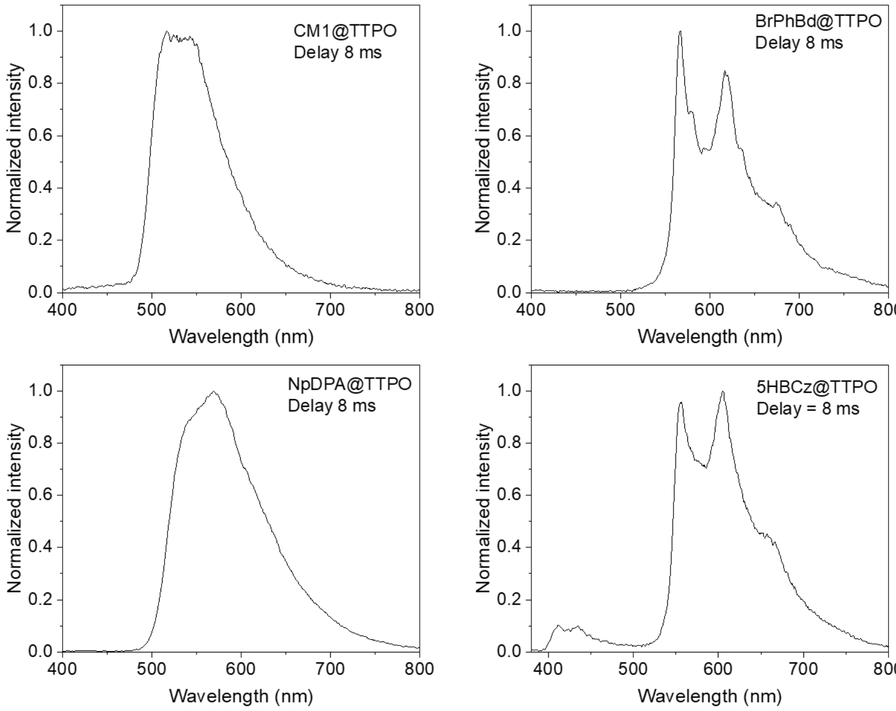

Supplementary Fig. 19 | Delayed spectra (8 ms) of guest@TTPO doping system (365 nm excitation).

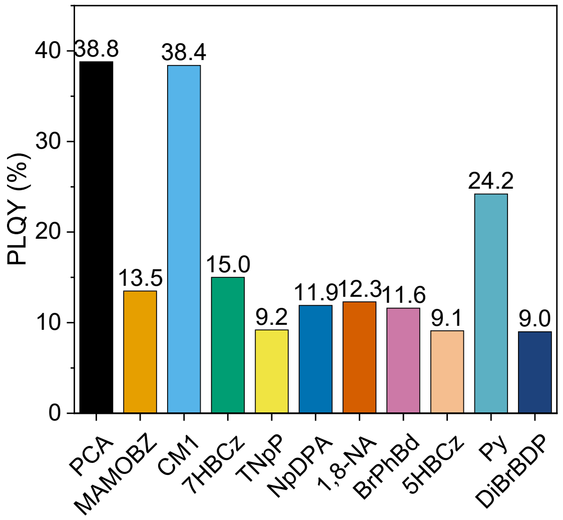

Supplementary Fig. 20 | Photoluminescence quantum yields (PLQYs) of guest@TTPO doping system (365 nm excitation).

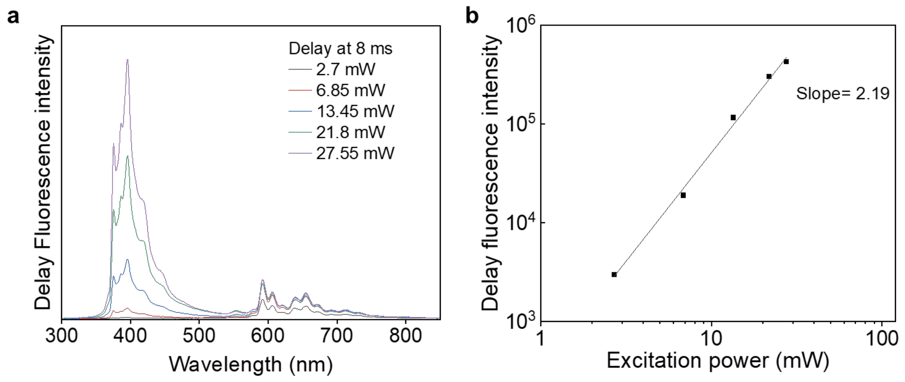

Supplementary Fig. 21 | Power-dependent experiments of Py@TTPO system. **a** Delay spectra measured at 8 ms under varying excitation intensities. **b** Logarithmic relationship between delayed fluorescence and excitation power intensity.

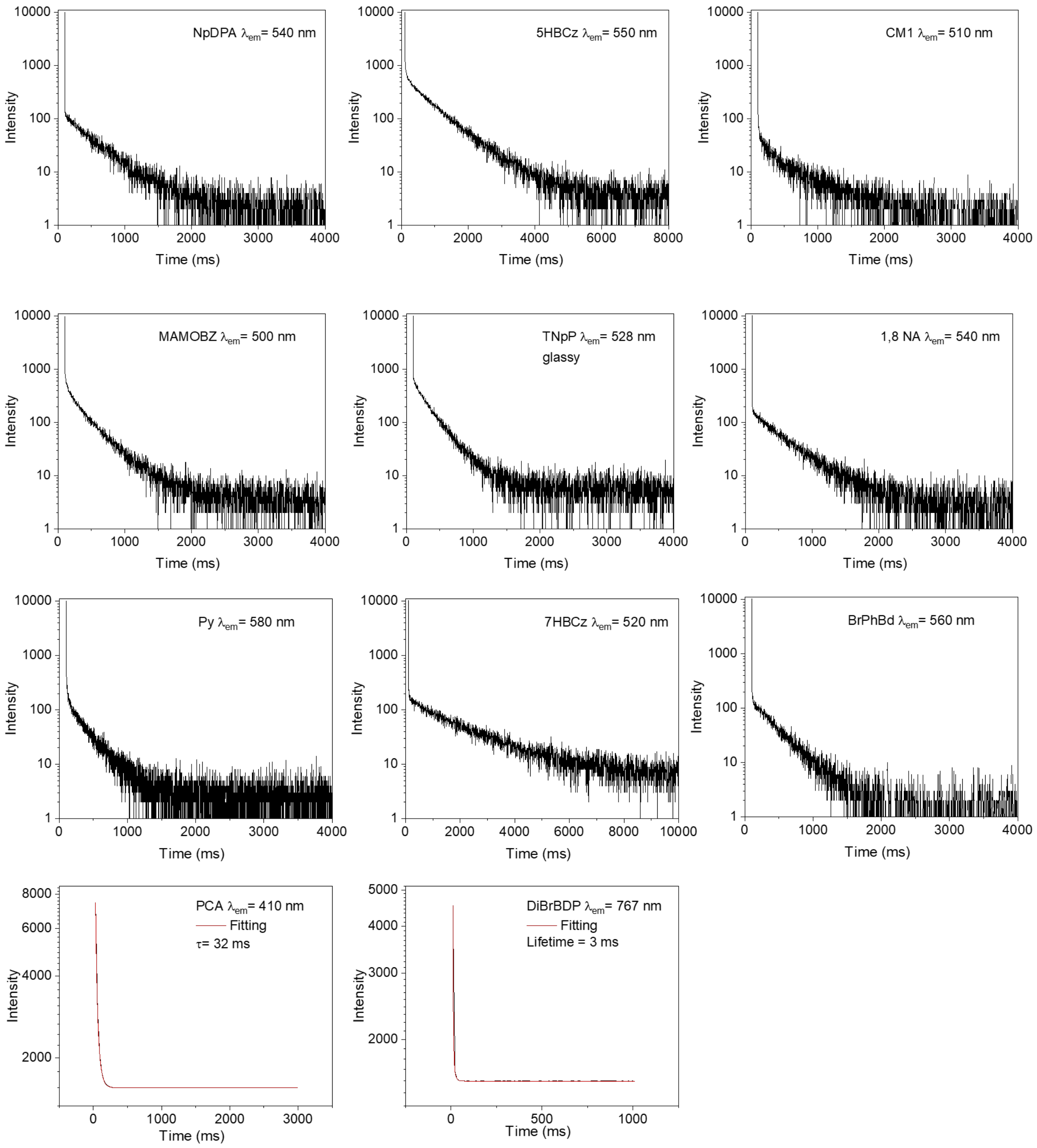

Supplementary Fig. 22 | Lifetime profiles of guest@TTPO doping system (365 nm excitation).

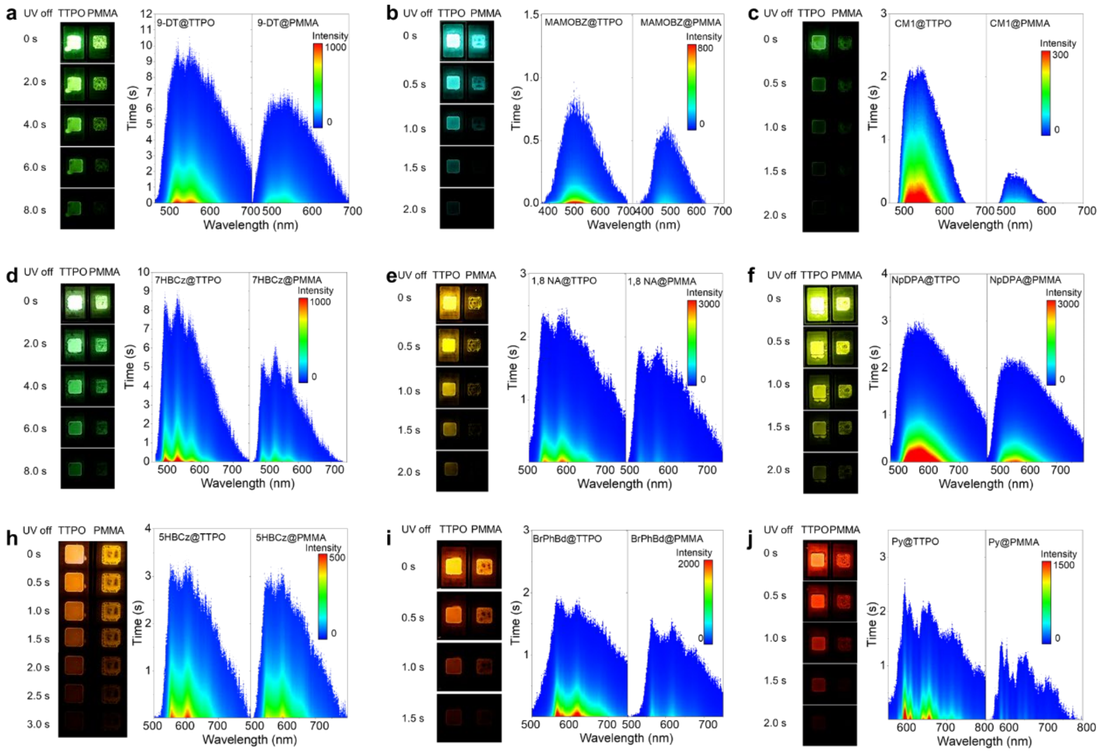

Supplementary Fig. 23 | TRES and afterglow images of dopants@TTPO vs dopants@PMMA.

`

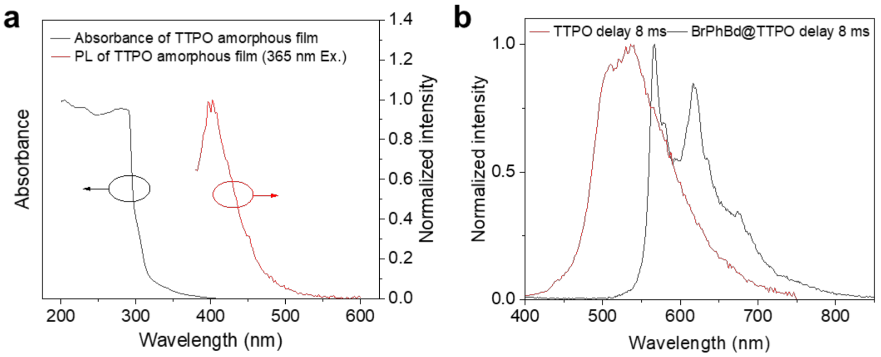

Supplementary Fig. 24 | a) Absorption and PL of TTPO amorphous film. b) The overlap of delay 8ms spectra of TTPO film and BrPhBd@TTPO doping system.

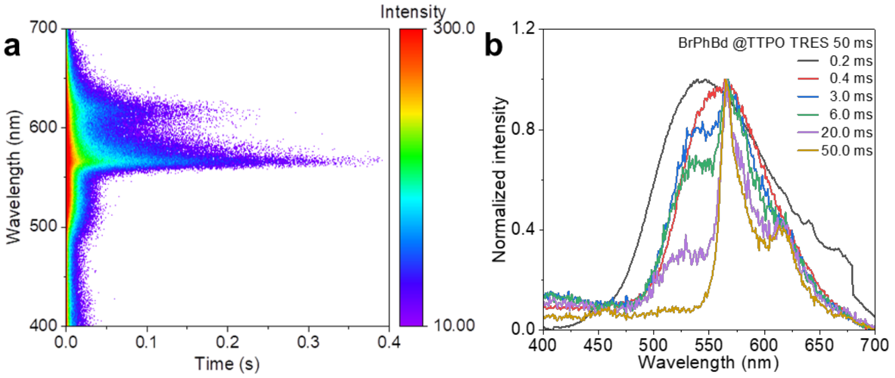

Supplementary Fig.25 | a) Microsecond TRES experiment of BrPhBd@TTPO doping system. b) Sliced emission spectra within 50 ms.

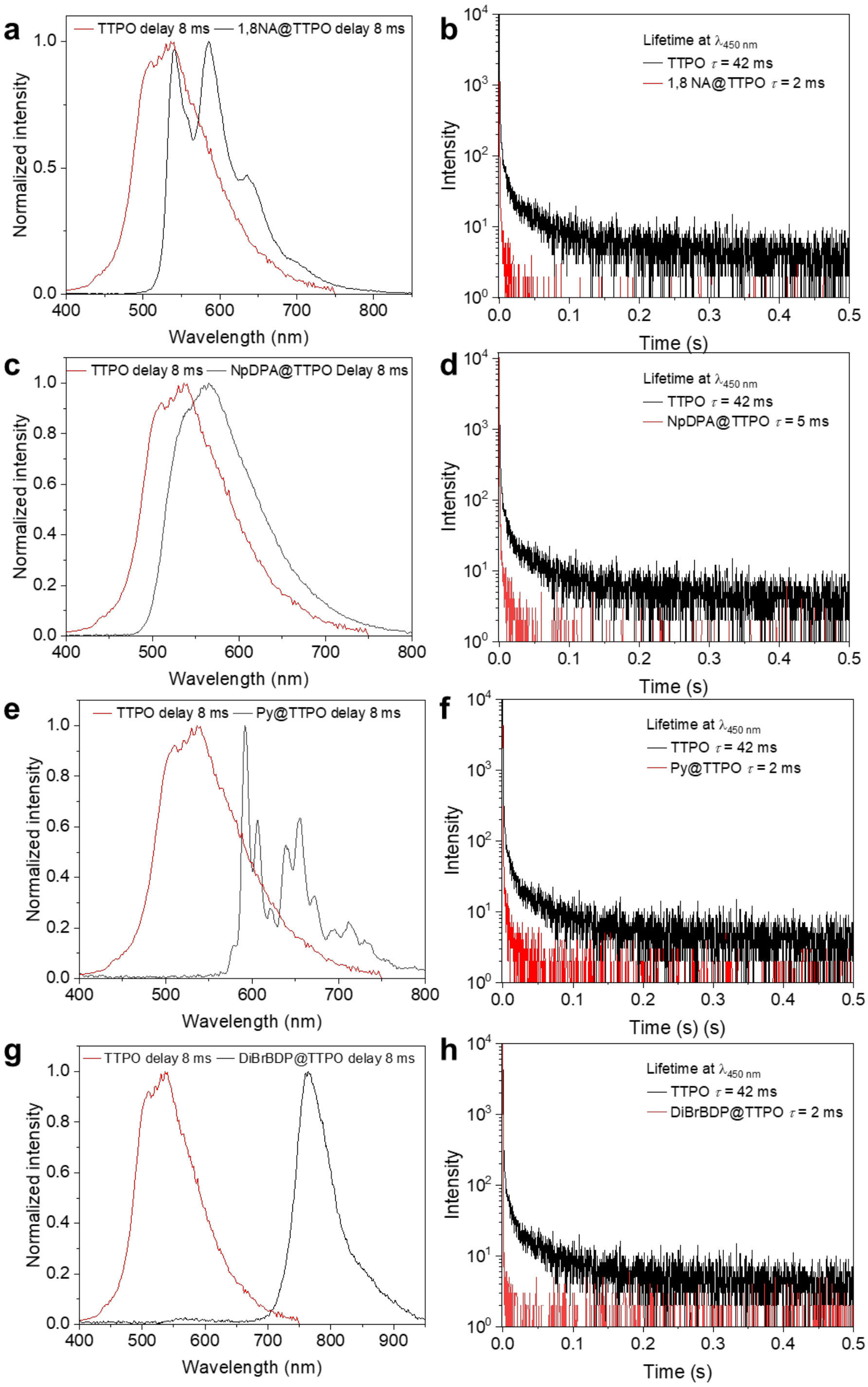

Supplementary Fig. 26 | a,c,e,g) Overlap of the delay 8 ms spectra of TTPO films and doping systems. b,d,f,h) The lifetime at 450 nm of TTPO film and dopants@TTPO doping system.

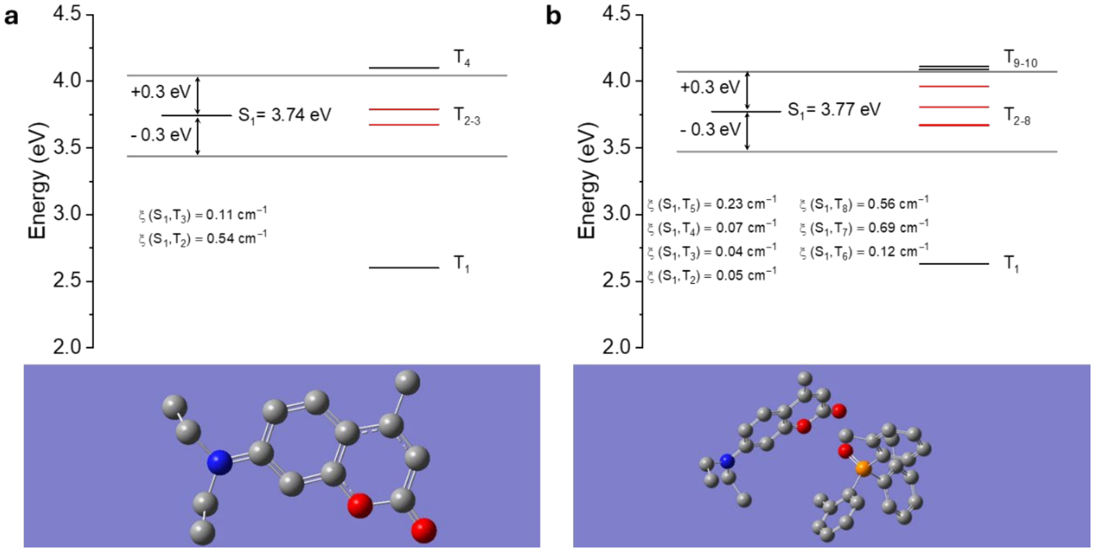

Supplementary Fig. 27 | DFT and TDDFT calculation results by using B3LYP/6-311g(d) basis set. SOCME calculation results by ORCA 5.0.4 using B3LYP/G functional and the DKH2 DKH-def2-TZVP basic set. **a** CM1 monomer. **b** CM1-TTPO pair (hydrogen atoms were omitted for clarity. Atoms: carbon (grey), phosphine (orange), nitrogen (blue), oxygen (red)) .

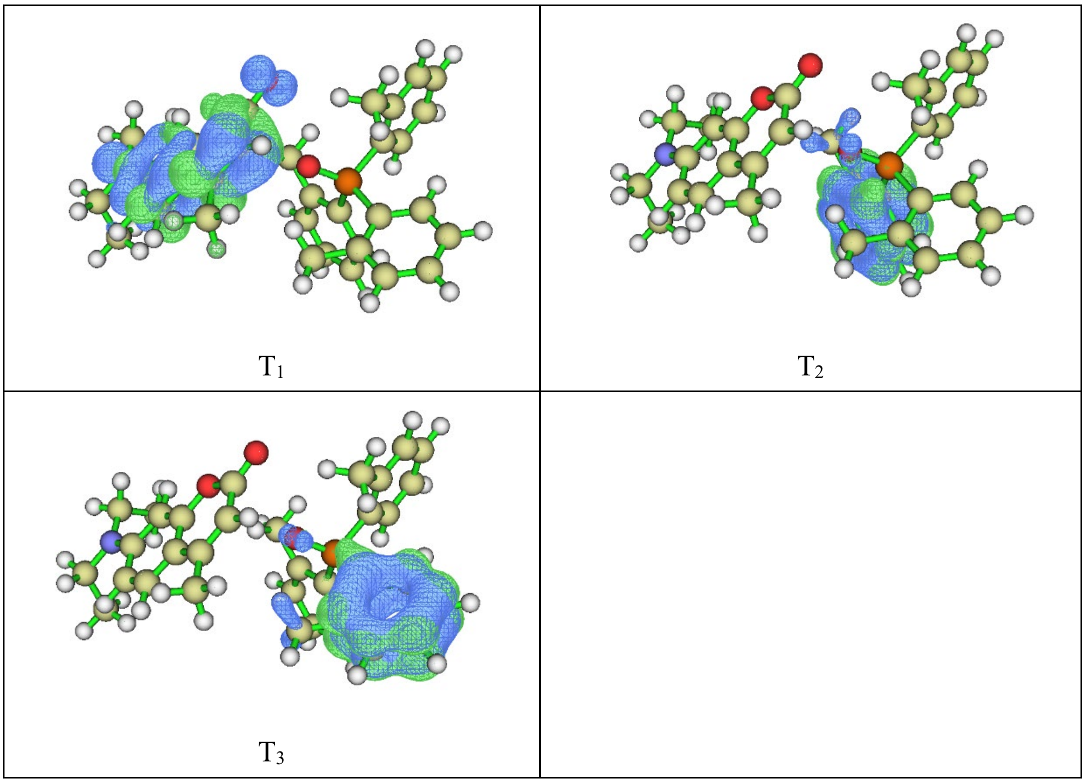

Supplementary Fig. 28 | Selected hole-electron (blue-green) diagrams of the T_1, T_2, T_3 states of CM1-TTPO pair in optimized conformations. Electron-hole densities of T_2 and T_3 were localized on the TTPO component and T_1 was on the guest (atoms carbon (yellow), hydrogen (grey), phosphine (orange), nitrogen (blue), oxygen (red)).

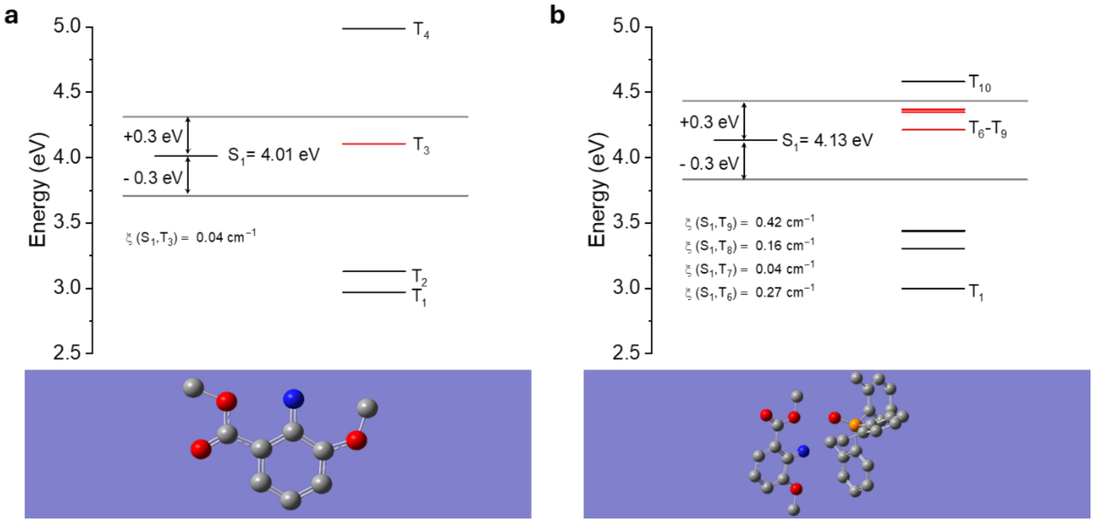

Supplementary Fig. 29 | DFT and TDDFT calculation results by using B3LYP/6-311g(d) basis set. SOCME calculation results by ORCA 5.0.4 using B3LYP/G functional and the DKH2 DKH-def2-TZVP basic set. **a** MAMOBZ monomer. **b** MAMOBZ-TTPO pair (hydrogen atoms were omitted for clarity. Atoms: carbon (grey), phosphine (orange), nitrogen (blue), oxygen (red)).

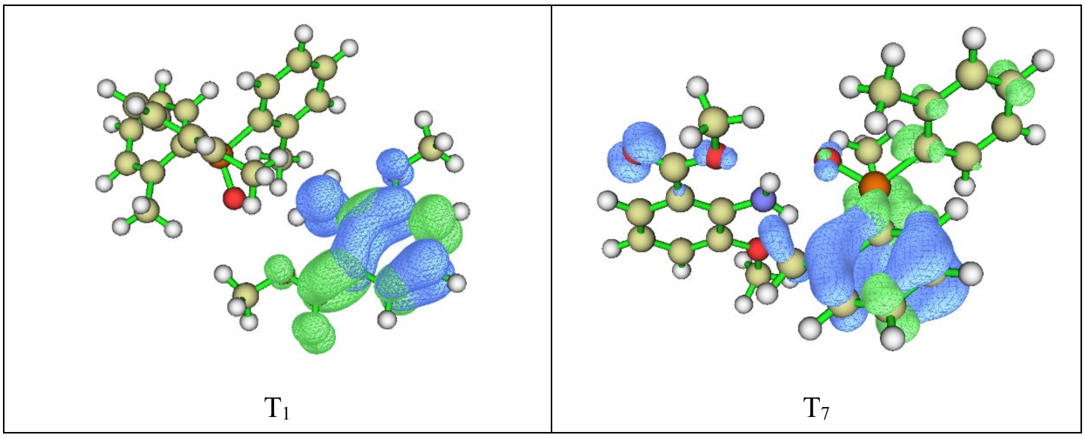

Supplementary Fig. 30 | Selected hole-electron (blue-green) diagrams of the T_1, T_7 states of MAMOBZ and TTPO in pair optimized conformations. Electron-hole density of T_7 was localized on the TTPO component and T_1 was on the guest (atoms carbon (yellow), hydrogen (grey), phosphine (orange), nitrogen (blue), oxygen (red))..

`

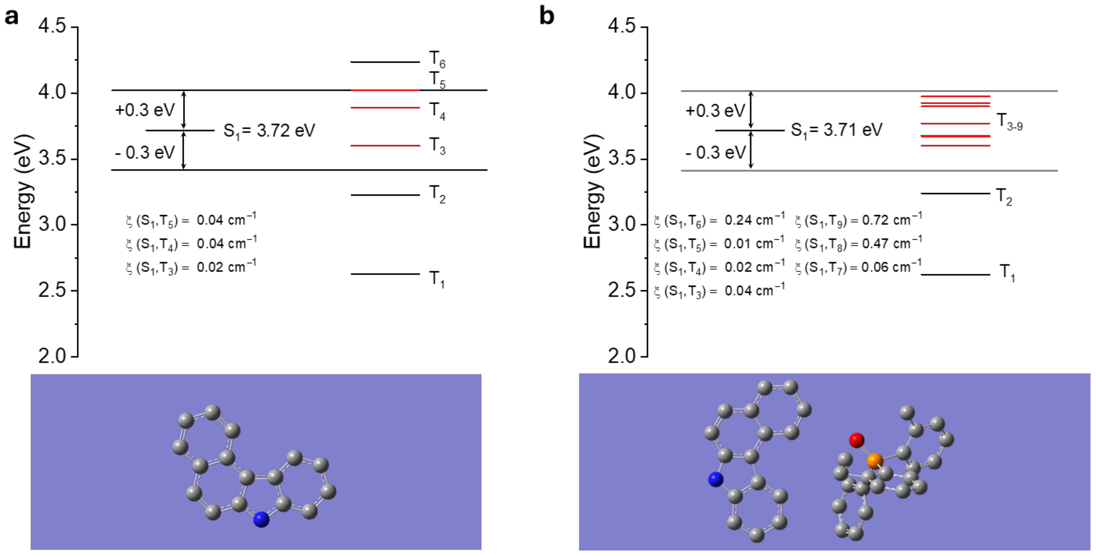

Supplementary Fig. 31 | DFT and TDDFT calculation results by using B3LYP/6-311g(d) basis set. SOCME calculation results by ORCA 5.0.4 using B3LYP/G functional and the DKH2 DKH-def2-TZVP basic set. **a** 7HBCz monomer. **b** 7HBCz-TTPO pair (hydrogen atoms were omitted for clarity. Atoms: carbon (grey), phosphine (orange), nitrogen (blue), oxygen (red)).

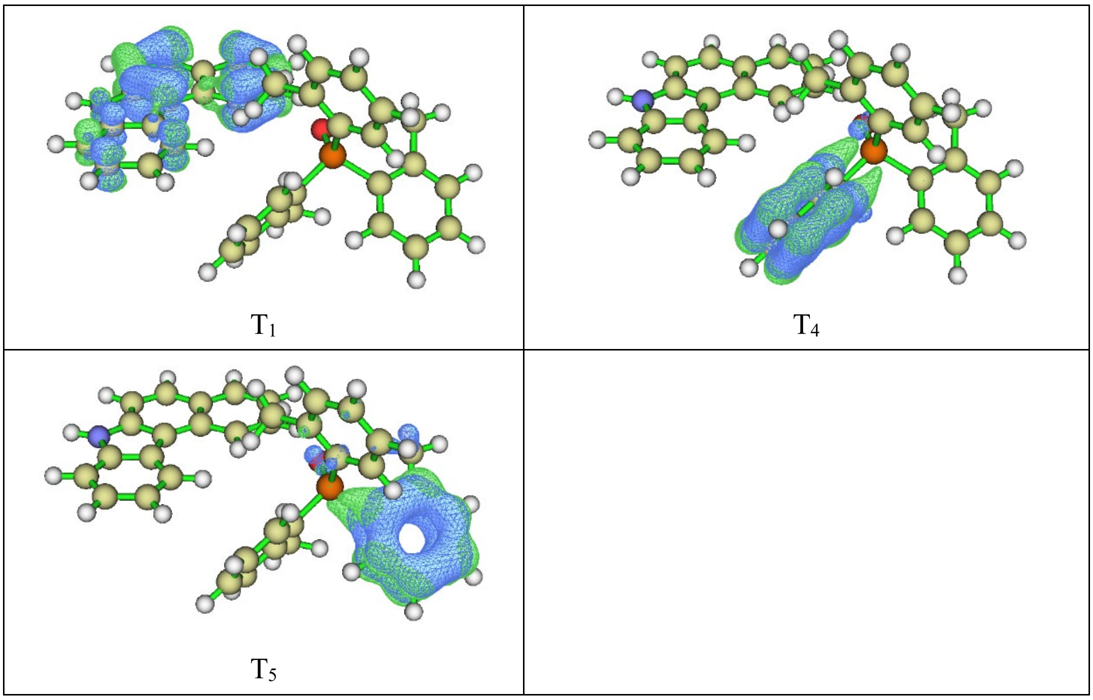

Supplementary Fig. 32 | Selected hole-electron (blue-green) diagrams of the T_1, T_4, T_5 states of 7HBCz-TTPO in optimized pair conformations. Electron-hole densities of T_4 and T_5 were localized on the TTPO component and T_1 was on the guest (atoms carbon (yellow), hydrogen (grey), phosphine (orange), nitrogen (blue), oxygen (red)).

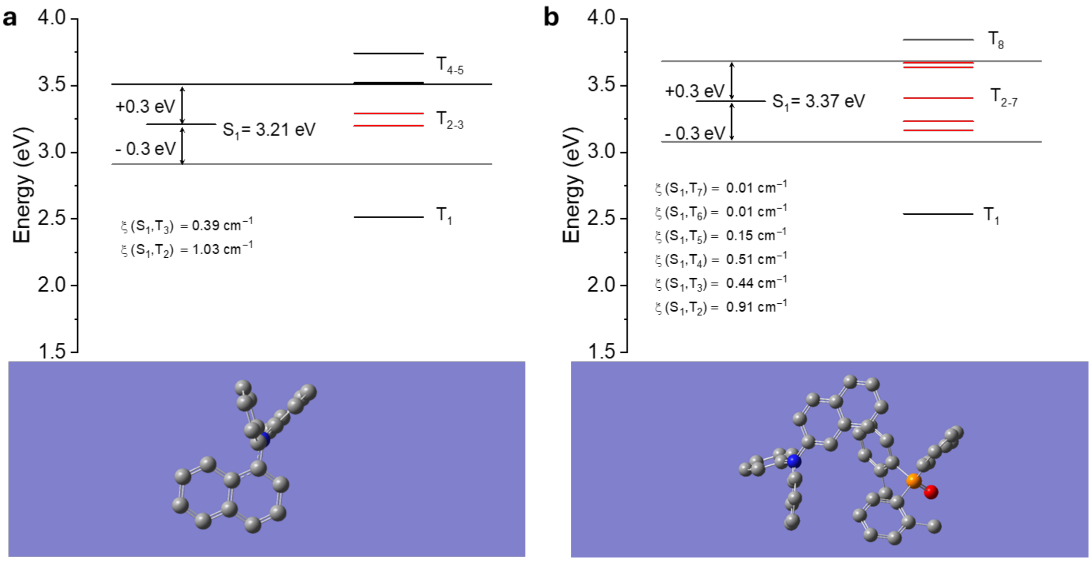

Supplementary Fig.33 | DFT and TDDFT calculation results by using B3LYP/6-311g(d) basis set. SOCME calculation results by ORCA 5.0.4 using B3LYP/G functional and the DKH2 DKH-def2-TZVP basic set. **a** NpDPA monomer. **b** NpDPA-TTPO pair (hydrogen atoms were omitted for clarity. Atoms: carbon (grey), phosphine (orange), nitrogen (blue), oxygen (red)).

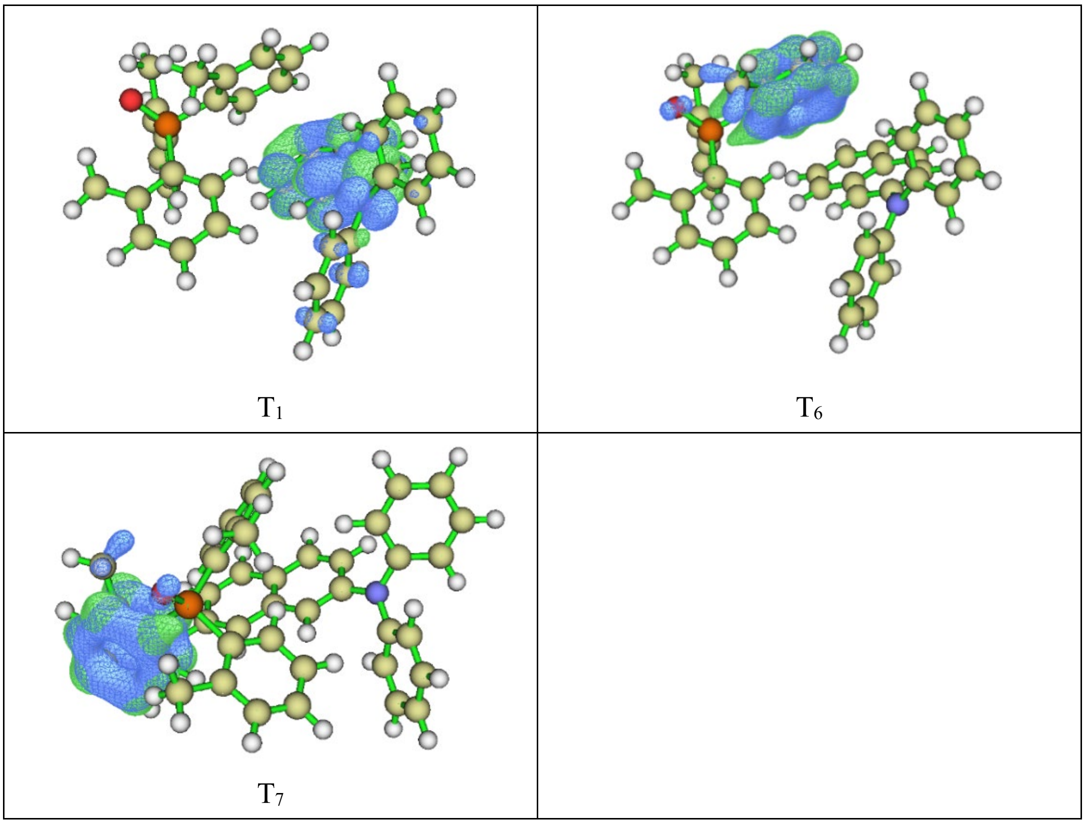

Supplementary Fig.34 | Selected hole-electron (blue-green) diagrams of the T_1, T_6, T_7 states of NpDPA and TTPO in pair optimized conformations. Electron-hole densities of T_6 and T_7 were localized on the TTPO component and T_1 was on the guest(atoms carbon (yellow), hydrogen (grey), phosphine (orange), nitrogen (blue), oxygen (red)).

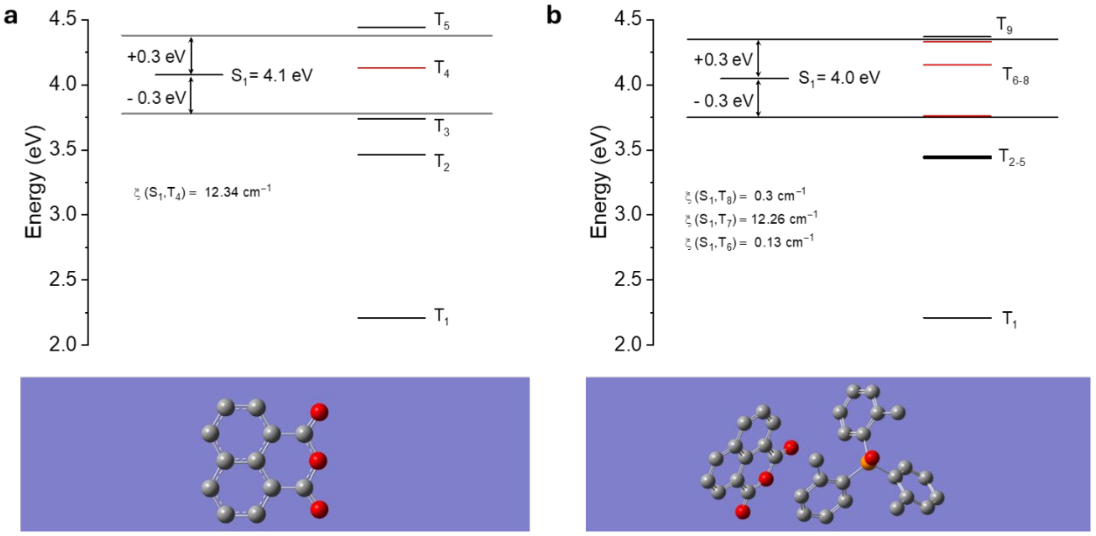

Supplementary Fig. 35 | DFT and TDDFT calculation results by using B3LYP/6-311g(d) basis set. SOCME calculation results by ORCA 5.0.4 using B3LYP/G functional and the DKH2 DKH-def2-TZVP basic set. **a** 1,8-NA monomer. **b** 1,8-NA-TTPO pair (hydrogen atoms were omitted for clarity. Atoms: Atoms: carbon (grey), phosphine (orange), oxygen (red)).

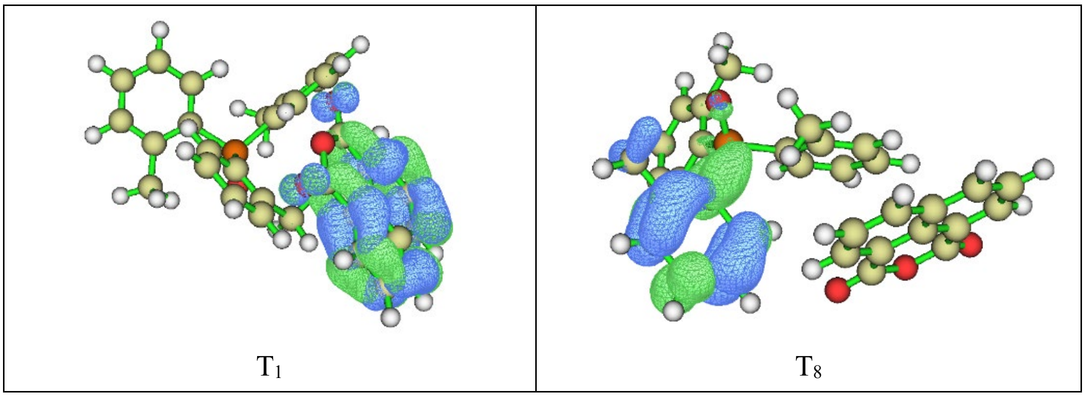

Supplementary Fig. 36 | Selected hole-electron (blue-green) diagrams of the T_1, T_8 states of 1,8-NA-TTPO in pair optimized conformations. Electron-hole density of T_8 was localized on the TTPO component and T_1 was on the guest (atoms carbon (yellow), hydrogen (grey), phosphine (orange), oxygen (red)).

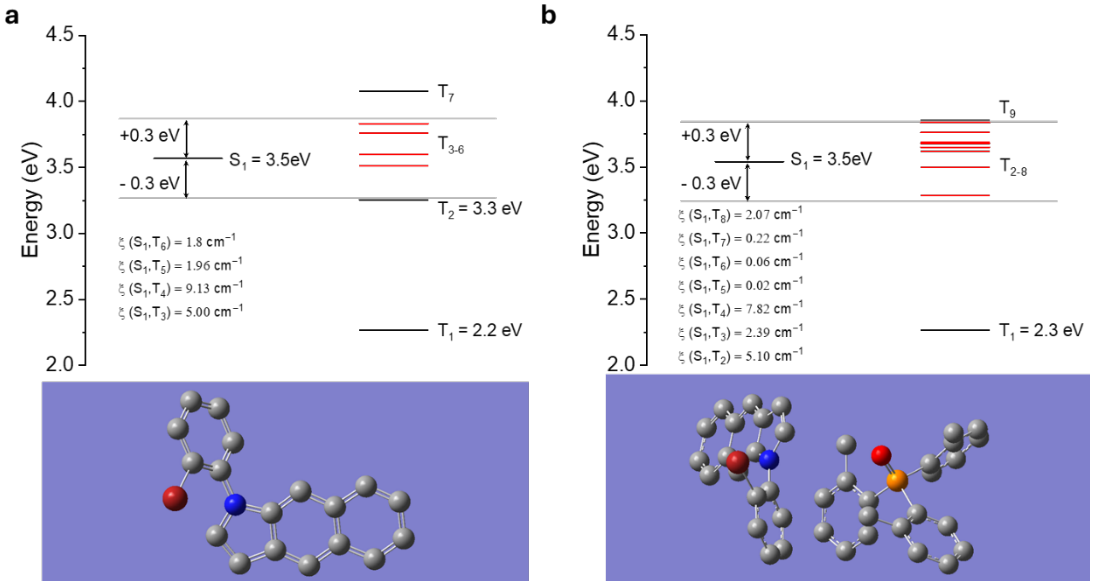

Supplementary Fig. 37 | DFT and TDDFT calculation results by using B3LYP/6-311g(d) basis set. SOCME calculation results by ORCA 5.0.4 using B3LYP/G functional and the DKH2 DKH-def2-TZVP basic set. **a** BrPhBd monomer. **b** BrPhBd-TTPO pair (hydrogen atoms were omitted for clarity. Atoms: carbon (grey), phosphine (orange), nitrogen (blue), oxygen (red)).

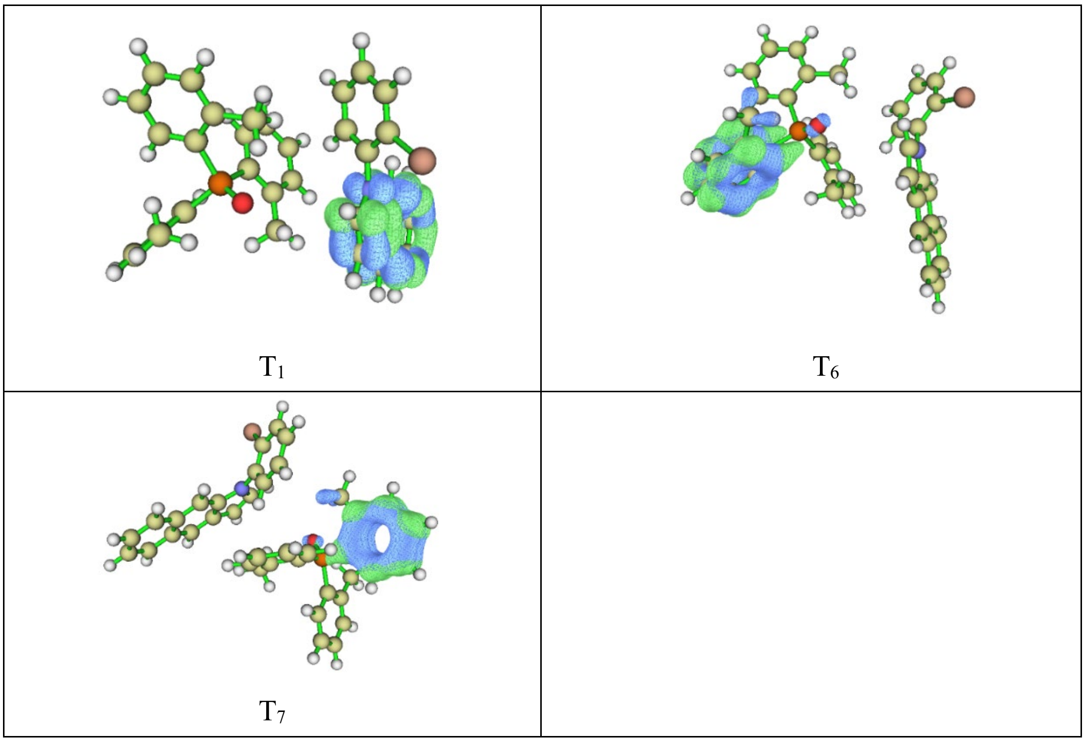

Supplementary Fig. 38 | Selected hole-electron (blue-green) diagrams of the T_1, T_6, T_7 states of BrPhBd -TTPO pair in optimized conformations. Electron-hole densities of T_6 and T_7 were localized on the TTPO component and T_1 was on the guest (atoms carbon (yellow), hydrogen (grey), phosphine (orange), nitrogen (blue), oxygen (red)).

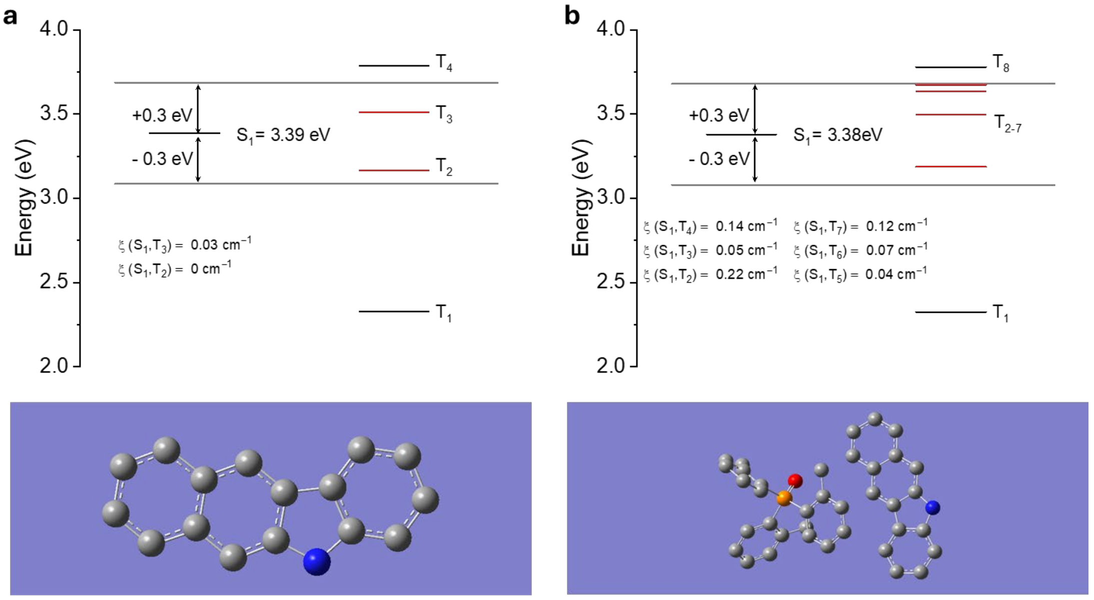

Supplementary Fig. 39 | DFT and TDDFT calculation results by using B3LYP/6-311g(d) basis set. SOCME calculation results by ORCA 5.0.4 using B3LYP/G functional and the DKH2 DKH-def2-TZVP basic set. **a**, 5HBCz monomer. **b**, 5HBCz-TTPO pair (hydrogen atoms were omitted for clarity. Atoms: carbon (grey), phosphine (orange), nitrogen (blue), oxygen (red)).

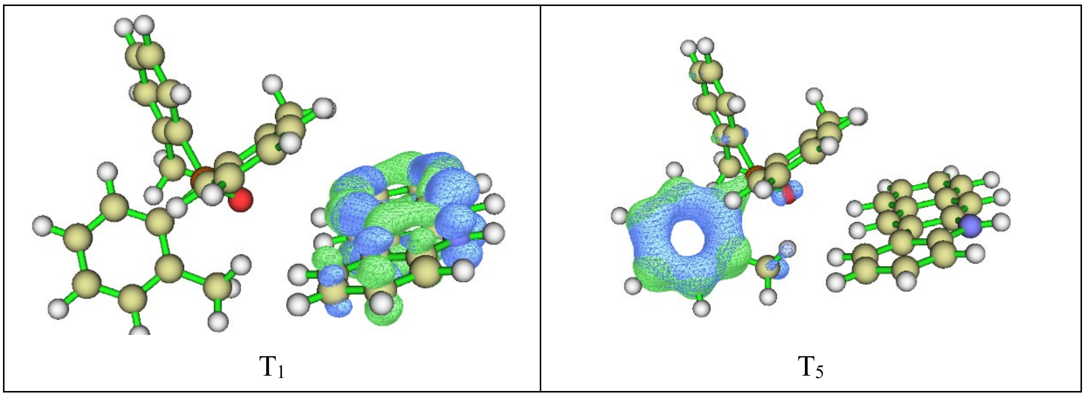

Supplementary Fig. 40 | Selected hole-electron (blue-green) diagrams of the T$_1$, T$_5$ states of 5HBCz-TTPO in pair optimized conformations. Electron-hole density of T$_5$ was localized on the TTPO component and T$_1$ was on the guest (atoms carbon (yellow), hydrogen (grey), phosphine (orange), nitrogen (blue), oxygen (red)).

`

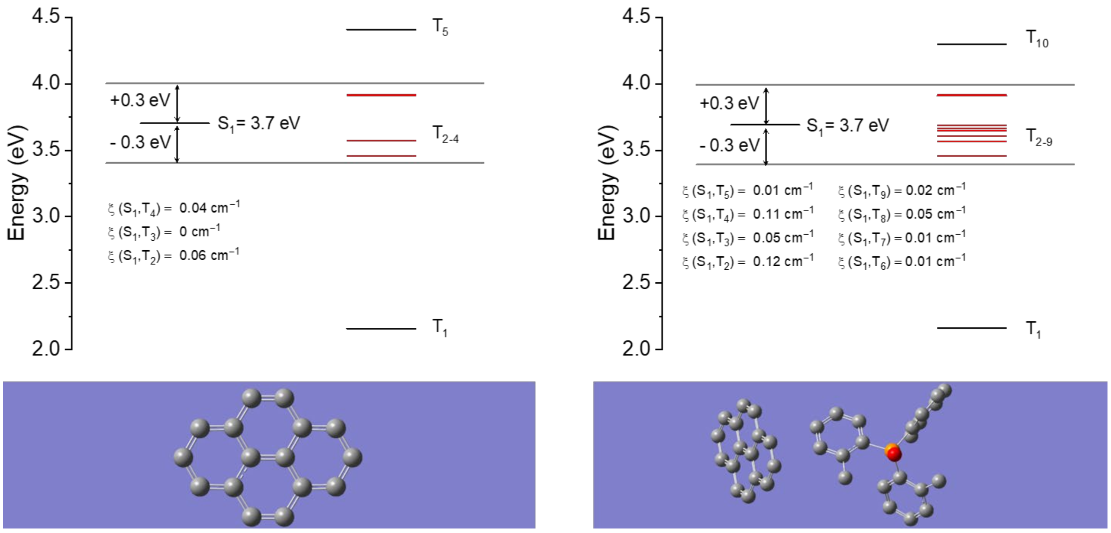

Supplementary Fig. 41 | DFT and TDDFT calculation results by using B3LYP/6-311g(d) basis set. SOCME calculation results by ORCA 5.0.4using B3LYP functional and the DKH-def2-TZVP basic set. **a**, Py monomer. **b**, Py-TTPO pair (hydrogen atoms were omitted for clarity. Atoms: carbon (grey), phosphine (orange), oxygen (red)).

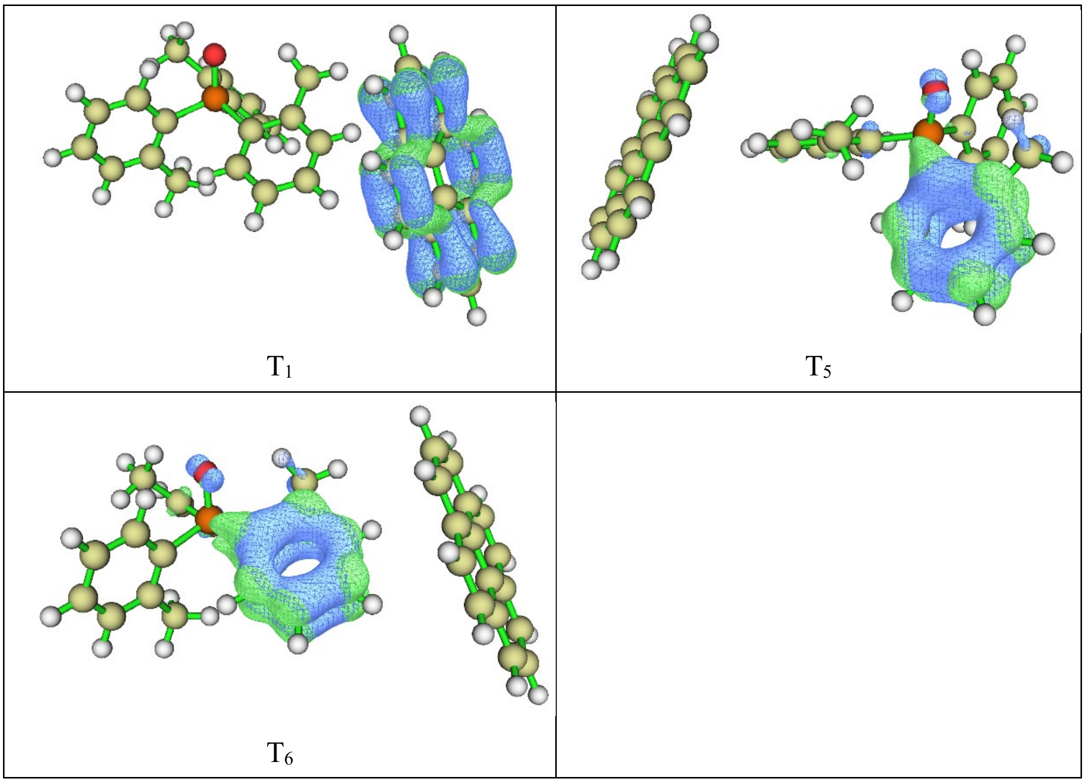

Supplementary Fig. 42 | Selected hole-electron (blue-green) diagrams of the T_1, T_5, T_6 states of Py-TTPO in pair optimized conformations. Electron-hole densities of T_5 and T_6 were localized on the TTPO component and T_1 was on the guest (atoms carbon (yellow), hydrogen (grey), phosphine (orange), oxygen (red)).

`

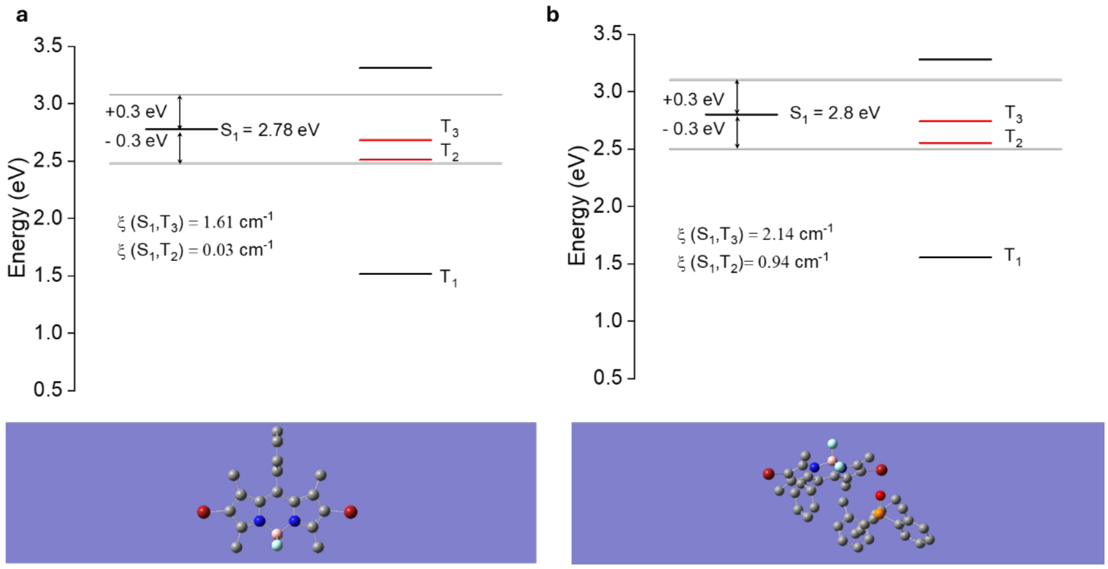

Supplementary Fig. 43 | DFT and TDDFT calculation results by using B3LYP/6-311g(d) basis set. SOCME calculation results by ORCA 5.0.4 using B3LYP/G functional and the DKH2 DKH-def2-TZVP basic set. **a**, DiBrBDP monomer. **b**, DiBrBDP-TTPO pair (hydrogen atoms were omitted for clarity. Atoms: carbon (grey), phosphine (orange), nitrogen (blue), oxygen (red), bromine (brownish red), boron (pink), fluorine (cyan)).

`

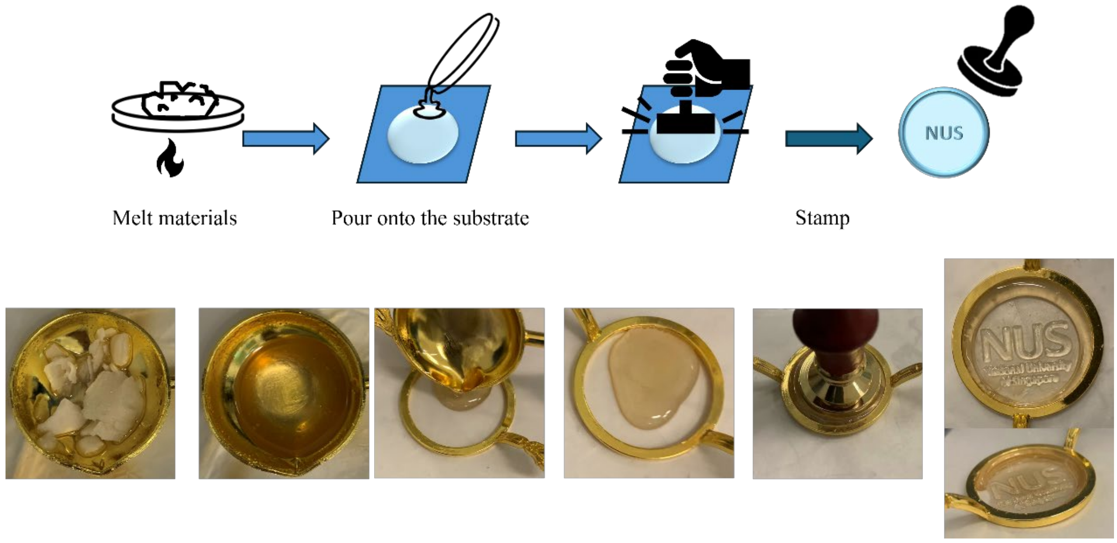

Supplementary Fig.44 | Procedure of hot pressing.

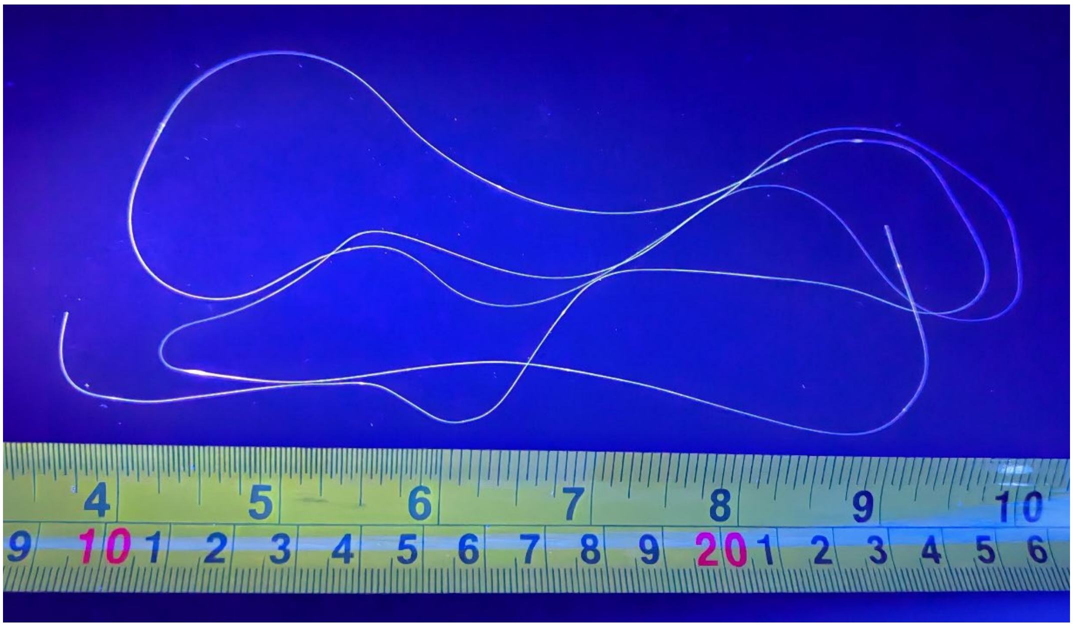

Supplementary Fig.45 | 1 meter fiber thermo-drawn from supercool liquid of TTPO.

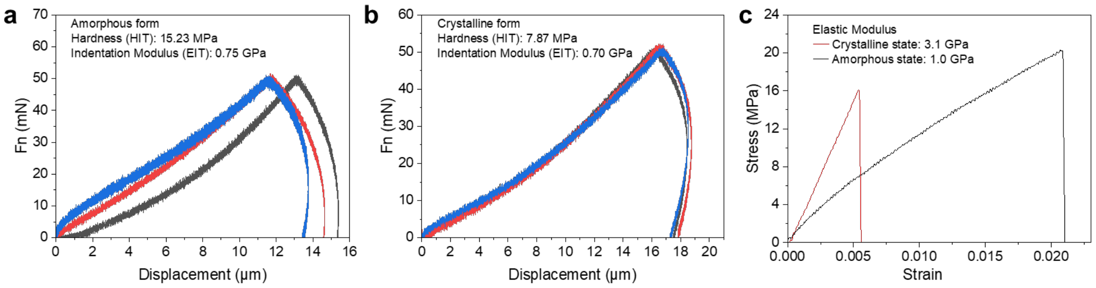

Supplementary Fig.46 | Mechanical properties of films and fibers. a,b) Mircoindentation measurement of glassy film and crystalline film. c) Tensile strength–displacement curve of amorphous fiber and crystalline fiber.

For the glassy film, the indentation hardness (HIT) and indentation modulus (EIT) were measured to be 15.23 MPa (mean value) and 0.75 GPa (mean value), respectively. After crystallization, the film became softer, with a reduced hardness of 7.87 MPa (mean value), while the indentation modulus remained at 0.70 GPa (mean value). The higher hardness in the amorphous state may be attributed to its disordered structure, which provides stronger intermolecular constraints. In the glassy state, molecules are arranged in a random, disordered manner, leading to a uniform distribution in different directions. This could help restrict structural slippage under external force and thereby enhancing hardness. In contrast, in the crystalline state, molecules adopt a more ordered arrangement, which introduces slip planes within the layers or crystal lattice. These slip planes may reduce the directional strength of intermolecular interactions, leading to lower hardness and increased susceptibility to deformation under stress.

For the fibers, the elastic modulus was measured at 1.0 GPa for the amorphous fiber and 3.1 GPa for the crystalline fiber, indicating that the amorphous fiber is significantly more flexible than its crystalline counterpart. Regarding mechanical strength and failure behavior, the crystalline fiber fractures at an early stage, failing at a strain of ~0.005 and a stress of 16 MPa, which suggests brittle failure, meaning the material cannot undergo significant deformation before breaking. In contrast, the amorphous fiber continues to deform up to ~0.02 strain before failing at a stress of 21 MPa, demonstrating higher ductility. The difference between amorphous and crystalline fibers can be explained by the molecular arrangement: the disordered structure of the amorphous fiber enables more uniform stress distribution, which contributes to its increased flexibility and greater strain accommodation, allowing it to sustain more deformation before failure.

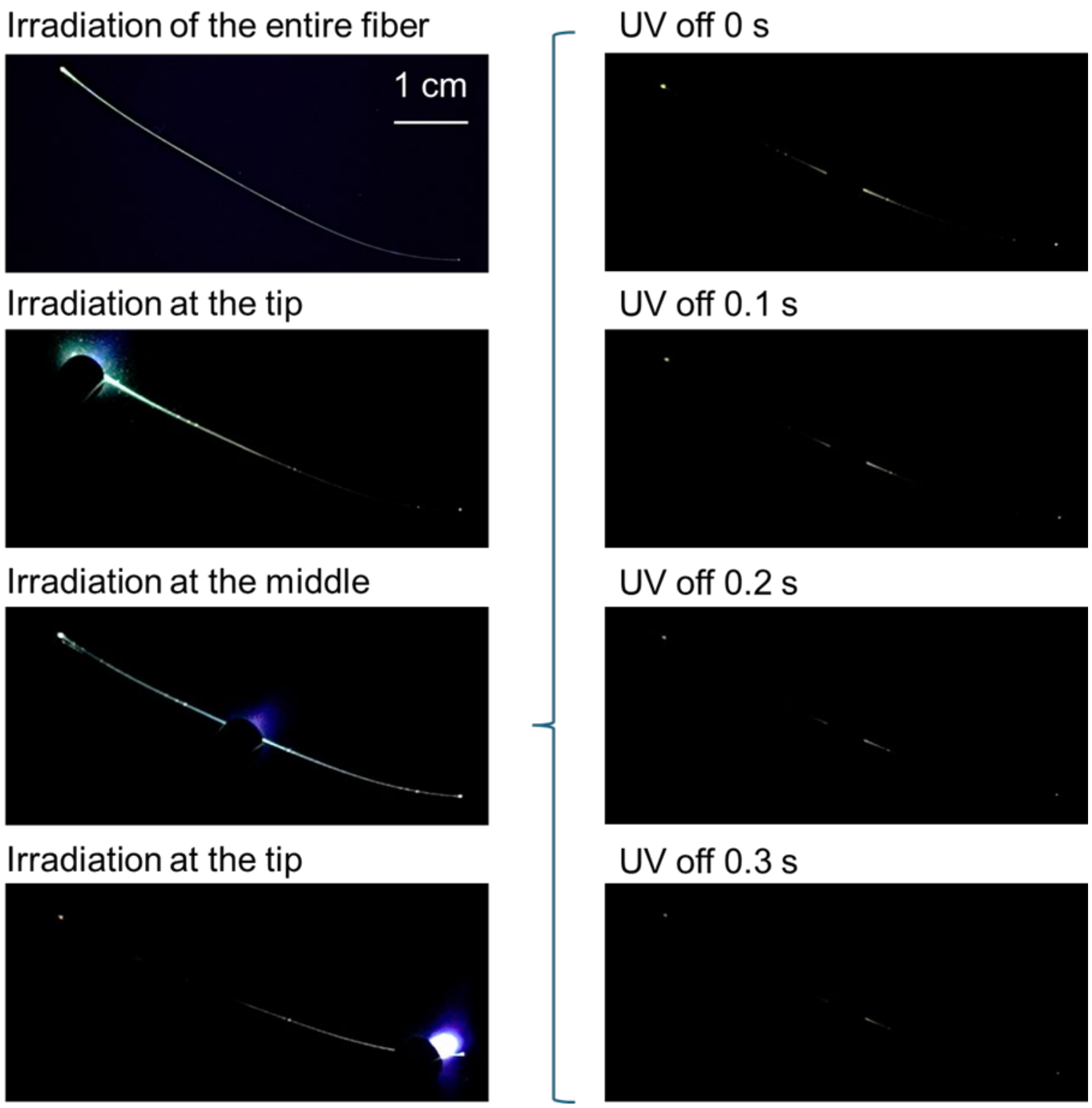

Supplementary Fig.47 | Demonstration of afterglow optical waveguide fiber.

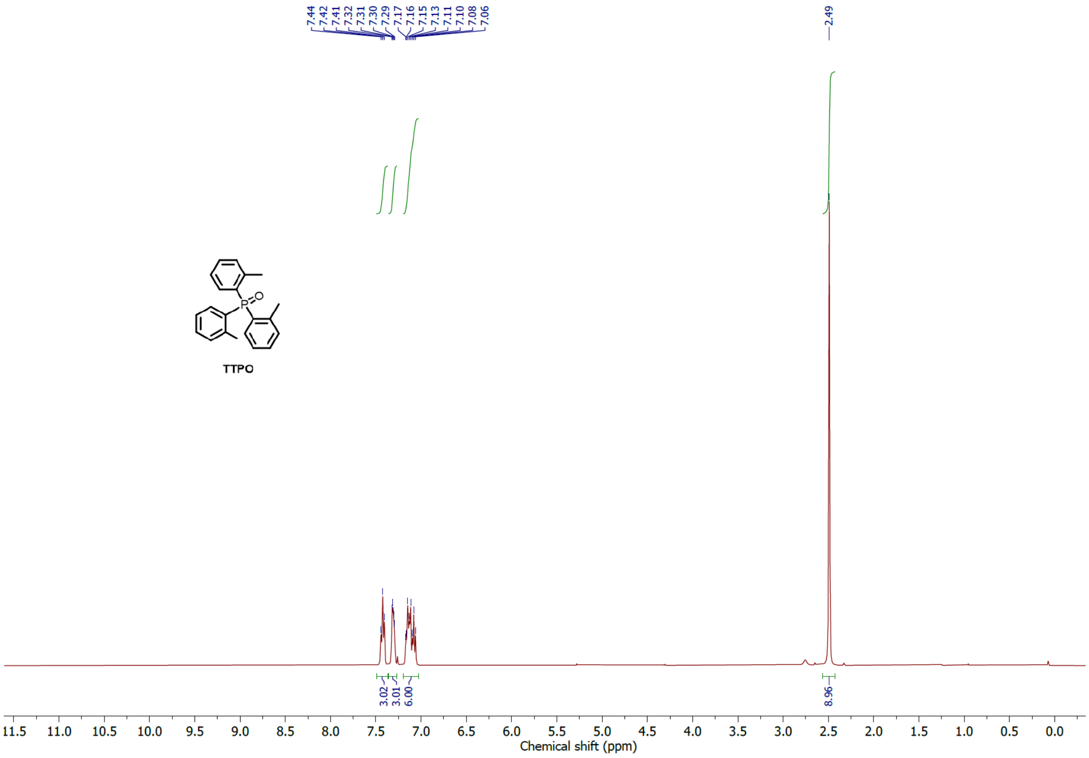

Supplementary Fig.48 | ^{1}H NMR spectra of TTPO in $CDCl_3$

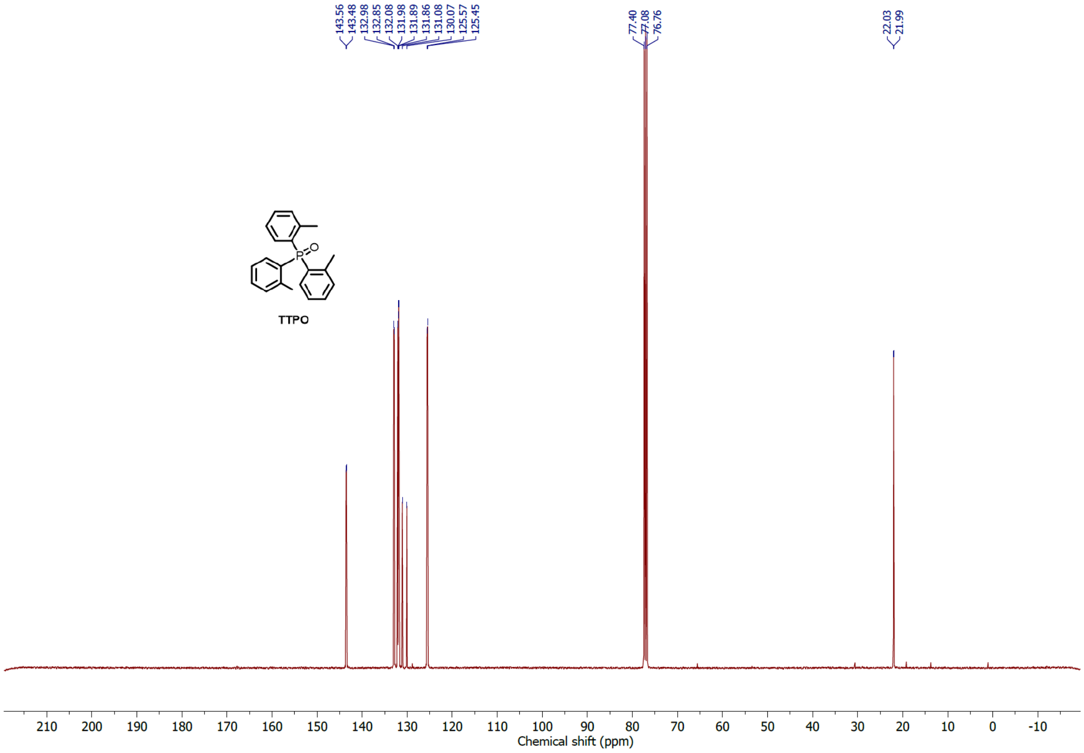

Supplementary Fig.49 | ^{13}C NMR spectra of TTPO in $CDCl_3$

`

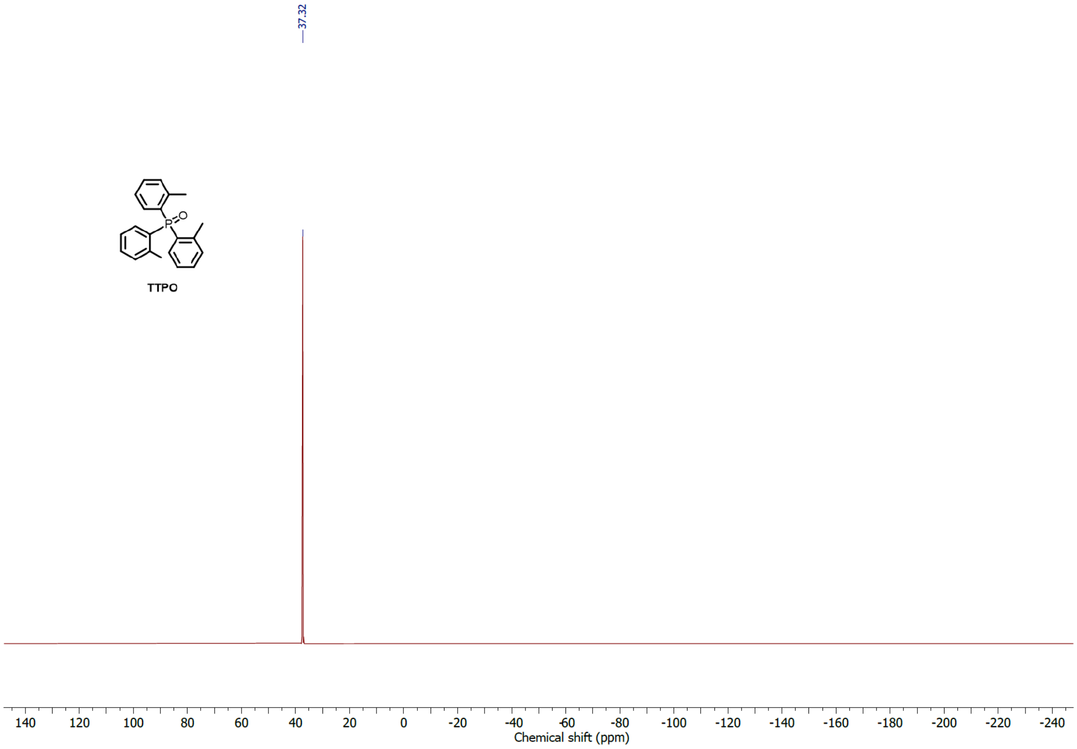

Supplementary Fig.50 | ^{31}P NMR spectra of TTPO in $CDCl_3$

`

Supplementary Table 1 Photophysical properties of different doping systems

	Φ_{PL} (%)	Φ_{phos} (%)	τ_p (ms)	Φ_{isc} $^{a)}$ (%)	k_p $^{b)}$ (s^{-1})	k_{nr} $^{c)}$ (s^{-1})
**MAMOBZ**	13.5	-	330	86.5	-	-
**CM1**	38.4	6.3	361	67.9	0.26	2.51
**7HBCz**	15.0	2.2	1695	87.2	0.01	0.58
**TNpP**	9.2	-	270	90.8	-	-
**NpDPA**	11.9	3.5	404	91.6	0.09	2.38
**1,8-NA**	12.3	2.7	450	90.4	0.07	2.16
**BrPhBd**	11.6	1.0	330	89.4	0.03	3.00
**5HBcZ**	9.1	1.8	795	92.7	0.02	1.23
**Py**	24.2	1.8	247	77.6	0.09	3.95
**DiBrBDP**	9.0	-	3	-	-	-

$^{a)}\Phi_{isc}=1-\Phi_F-\Phi_{IC} \approx 1-\Phi_{PL}+\Phi_{phos}$; $^{b)}$ $k_p=\Phi_{phos}/(\Phi_{isc}\,\tau_p)$; $^{c)}$ $k_{nr}=1/(\tau_p\,k_p)$

Where Φ_{PL} is the total photoluminescence quantum yield. Φ_{phos} is the phosphorescence quantum yield. τ_p is the lifetime of phosphorescence. Φ_{IC} is the quantum efficiencies of internal conversion. Φ_{ISC} is the quantum efficiencies of intersystem crossing. k_p is the rate constant of phosphorescence. k_{nr} is the rate constant of non-radiative decay[7].

Supplementary Table 2 Crystal data and structure refinement of TTPO

**Formula**	$C_{21}H_{21}OP$	**ρ_{calc}/g cm^{-3}**	1.226
**Formula weight**	320.35 g/mol	**Z**	4
**Temperature / K**	100 K	**Absorption coefficient**	1.402
**Crystal system**	Monoclinic	**F (000)**	680.0
**Space group**	$P2_1/c$	**Crystal size/mm^3**	0.161 × 0.114 × 0.076
**a / Å**	15.9602(5)	**Wavelength / Å**	1.54178
**b / Å**	7.7521(2)	**Reflections collected**	0.0422 (2734)
**c / Å**	14.2751(4)	**Data/ restraints/ parameters**	3065/ 277/ 276
**α /°**	90	**Independent reflections**	3065 [R_{int} =0.0497, R_{sigma} = 0.0296]
**β / °**	100.661(2)	**Goodness-of-fit on F2**	1.100
**γ / °**	90	**R_1, [a] wR_2 [b] [I≥2σ (I)]**	R_1 = 0.0422, wR_2 = 0.0942
**Volume / Å^3**	1735.70(9)	**R_1, wR_2 [all data]**	R_1 = 0.0485, wR_2 = 0.0997

`

References

1. Gaussian 09, Revision D.01, J. Frisch, G. W. Trucks, H. B. Schlegel, G. E. Scuseria, M. A. Robb, J. R. Cheeseman, G. Scalmani, V. Barone, B. Mennucci, G. A. Petersson, H. Nakatsuji, M. Caricato, X. Li, H. P. Hratchian, A. F. Izmaylov, J. Bloino, G. Zheng, J. L. Sonnenberg, M. Hada, M. Ehara, K. Toyota, R. Fukuda, J. Hasegawa, M. Ishida, T. Nakajima, Y. Honda, O. Kitao, H. Nakai, T. Vreven, J. A. Montgomery, Jr., J. E. Peralta, F. Ogliaro, M. Bearpark, J. J. Heyd, E. Brothers, K. N. Kudin, V. N. Staroverov, T. Keith, R. Kobayashi, J. Normand, K. Raghavachari, A. Rendell, J. C. Burant, S. S. Iyengar, J. Tomasi, M. Cossi, N. Rega, J. M. Millam, M. Klene, J. E. Knox, J. B. Cross, V. Bakken, C. Adamo, J. Jaramillo, R. Gomperts, R. E. Stratmann, O. Yazyev, A. J. Austin, R. Cammi, C. Pomelli, J. W. Ochterski, R. L. Martin, K. Morokuma, V. G. Zakrzewski, G. A. Voth, P. Salvador, J. J. Dannenberg, S. Dapprich, A. D. Daniels, O. Farkas, J. B. Foresman, J. V. Ortiz, J. Cioslowski, and D. J. Fox, Gaussian, Inc., Wallingford CT, 2013.
2. F. Neese, *WIREs Comput. Mol. Sci.* **2012**, *2*, 73;
3. F. Neese, *WIREs Comput. Mol. Sci.* **2018**, *8*, e1327;
4. F. Neese, F. Wennmohs, U. Becker, C. Riplinger, *J. Chem. Phys.* **2020**, *152*, 224108.
5. T. Lu; F. Chen, *J. Comput. Chem.* **2012**, *33*, 580.
6. M.R. Ivancevic, J.A. Wisch, Q.C. Burlingame, B.P. Rand, Y.L. Loo, *Adv. Mater.,***2024**, *36*, 2402478.
7. H. Ma, L. Fu, X. Yao, X. Jiang, K. Lv, Q. Ma, H. Shi, Z. An, W. Huang, *Nat. Commun.* **2024**, *15*, 3660.
