# Supplementary material for: Full-color processible afterglow organic small molecular glass
Source: Nat Commun. 2025 May 15;16:4526. doi: 10.1038/s41467-025-59787-y (PMC12081614; doi:10.1038/s41467-025-59787-y)
Supplement: Supplementary file 2 — Description of Additional Supplementary Files [file 41467_2025_59787_MOESM2_ESM.pdf]

Description of Additional Supplementary Files

File name: Supplementary Data 1
Description: Optimized structures
